# Global, regional, and national trends of syphilis from 1990 to 2019: the 2019 global burden of disease study

**DOI:** 10.1186/s12889-023-15510-4

**Published:** 2023-04-24

**Authors:** Yu-Ting Tao, Teng-Yu Gao, Hao-Yang Li, Yu-Tong Ma, Hui-Jun Li, Chen-Yang Xian-Yu, Nian-Jia Deng, Chao Zhang

**Affiliations:** Center for Evidence-Based Medicine and Clinical Research, Taihe Hospital, Hubei University of Medicine, No.32, Renmin South Road, Shiyan, 442000 Hubei China

**Keywords:** Syphilis, Global burden of disease, Incidence, Mortality, Disability-adjusted life year

## Abstract

**Background:**

Syphilis is a sexually transmitted disease caused by *Treponema pallidum*, and the infection source is syphilis patients. This study aimed to estimate the incidence, mortality rate, and disability-adjusted life years (DALYs) of syphilis to improve the understanding of the current global situation of syphilis.

**Methods:**

This study collected data on syphilis incidence, mortality, and DALYs from the 2019 Global Burden of Disease database.

**Results:**

The global number of incident cases and age-standardized incidence rate (ASIR) increased from 8,845,220 (95% UI: 6,562,510–11,588,860) in 1990 to 14,114,110 (95% UI: 10,648,490–18,415,970) in 2019 and 160.03/100,000 persons (95% UI: 120.66–208.1) to 178.48/100,000 persons (95% UI: 134.94–232.34), respectively. The estimated annual percentage change (EAPC) in the ASIR was 0.16 (95% CI: 0.07–0.26). The EAPC in the ASIR associated with high and high-middle sociodemographic indices increased. The ASIR increased among males but decreased among females, and the incidence peaked among males and females between the ages of 20 and 30 years. The EAPCs in the age-standardized death rate and age-standardized DALY rate decreased.

**Conclusions:**

The incidence and ASIR of syphilis increased worldwide from 1990 to 2019. Only the regions with high and high-middle sociodemographic indices showed an increase in the ASIR. Moreover, the ASIR increased among males but decreased among females. The age-standardized death rate and DALY rate both declined worldwide. The increase in the global ASIR of syphilis is a challenge.

**Supplementary Information:**

The online version contains supplementary material available at 10.1186/s12889-023-15510-4.

## Background

Syphilis is a sexually transmitted disease caused by *Treponema pallidum* (TP) [1], and the infection source is syphilis patients. Syphilis is highly infectious, and its early symptoms are chancroid and syphilis rash [2]. After contracting syphilis, specific TP antibodies develop in the serum with indefinite positive reactions [3].

More than 5 million new cases [4] of syphilis are reported worldwide each year. Epidemiological studies have reported that the incidence of syphilis among males has more than tripled in the United States since 2000, leading to a sharp increase in early cases [5]. Early in 2012, the World Health Organization reported that the number of infected people aged 15–49 years had reached 17.7 million [6]. In regions with severe poverty and poor development, syphilis is inevitably widespread, given the prevalence of sexually transmitted diseases. Syphilis emerged as early as the sixteenth century [7] and has continued to plague humans. To control syphilis, the greatest challenge is preventing transmission. Currently, perceptions and behaviours are more open, and statistical data are needed to determine the current syphilis situation.

The syphilis population according to the Global Burden of Disease (GBD) reports for the last 30 years, from 1990 to 2019, was classified by region, sociodemographic index (SDI), age, and sex, and the incidence, mortality rate, disability-adjusted life years (DALYs), and changing trends of syphilis were statistically estimated. This study can provide national health organizations and syphilis researchers with a better understanding of the global situation of syphilis and effective data to guide the management and prevention of syphilis.

## Methods

### Data sources

The Global Health Data Exchange (GHDx) query tool (http://ghdx.healthdata.org/gbd-results-tool) [8] was used to obtain data including the annual incidence, mortality rate, and DALYs for syphilis from 1990 to 2019. The demographic data of the syphilis population obtained were nationality, sex, and age. Two hundred four countries and territories around the world were divided into 21 subregions [9] to facilitate in-depth estimations of the trends in different regions. In addition, this study retrieved the SDIs for 204 countries and regions for the last 30 years. The SDIs were categorized into low, low-middle, middle, high-middle, and high, and their association with morbidity, mortality, and DALYs was estimated. The SDI denotes the overall capacity of a country and incorporates income per capita, educational attainment, and total fertility rates, among other factors [10]. The global disease burden of syphilis was determined with comparison charts and maps.

### Statistical analysis

**Fig. 1:**
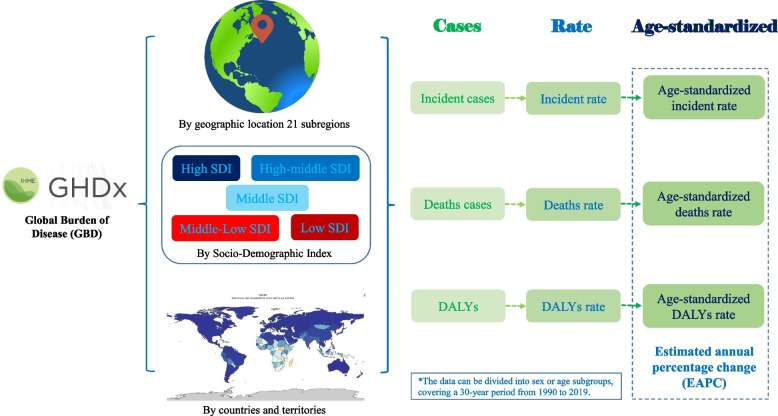
Visualized flow chart for describing methods

In this study, the incidence and mortality rates of syphilis were evaluated based on the annual age-standardized incidence rate (ASIR), age-standardized death rate (ASDR), and age-standardized DALY rate to prevent the influence of age on the data. The most obvious advantages of the ASIR are that it eliminates the effect of different ages and provides more impartial criteria to explain the epidemic characteristics of syphilis. DALYs are the sum of years lived with disability (YLD) and years of life lost (YLL) [11] due to premature mortality. The ASR (age-standardized incidence/death/DALY rate) (per 100,000 population) is the sum of the products of the specific age ratios (${a}_{i}$) of the age groups $(i)$ and the sizes (or weights) (${w}_{i}$) of the reference standard population groups $(i)$ divided by the sum of the sizes (or weight) of the reference populations; it is represented as $\mathrm{ASR}=\frac{{\sum }_{i=1}^{A}{a}_{i}{w}_{i}}{{\sum }_{i=1}^{A}{w}_{i}}\times 100, 000$ [12]. The data for the cases and rates were limited by a 95% uncertainty interval (UI) [13]. With the known ASR in the database, the estimated annual percentage changes (EAPCs) and their 95% confidence intervals (CIs) can be obtained by the following two regression equations. The formula is y = α + βx + ɛ, $EAPC=\left(\frac{{\widehat{ASR}}_{x+1}-{\widehat{ASR}}_{x}}{{\widehat{ASR}}_{x}}\right)\cdot 100=\left(\frac{{\widehat{ASR}}_{x+1}}{{\widehat{ASR}}_{x}}-1\right)\cdot 100=\left(\frac{{e}^{\widehat{y}+\widehat{\beta }}\left(x+1\right)}{{e}^{\widehat{y}+\widehat{\beta }}\cdot x}-1\right)\cdot 100=\left(exp\left(\beta \right)-1\right)\times 100$ [14], where y represents ln (ASR), and x is expressed as the calendar year. We obtained the ASR directly from the database, so plugging x into the first formula can calculate β. Then, the EAPC was calculated by substituting β into the second simplified formula. When the EAPC value and lower limit of the 95% CI are positive, the ASR tends to increase; higher values are associated with more rapid increments in the ASR. When the EAPC value and upper limit of the 95% CI are negative, the ASR tends to decrease; higher absolute values are associated with a more rapid decline in the ASR. When the EAPC value is close to 0 and the maximum and minimum 95% CIs are on either side of 0, the ASR tends to be stable. To more intuitively estimate the changing ASR trends, this study also evaluated the changing situations in different parts of the world through mapping. Scatter plots were used to determine the relationship between the ASR and SDI, with positive and negative ρ (Pearson’s coefficient) values representing positive and negative correlations, respectively. *P* values less than 0.05 denoted statistical significance. In this study, a visual flow chart (Fig. 1) was drawn to present the research process more clearly.

## Results

### Global burden of syphilis

**Fig. 2:**
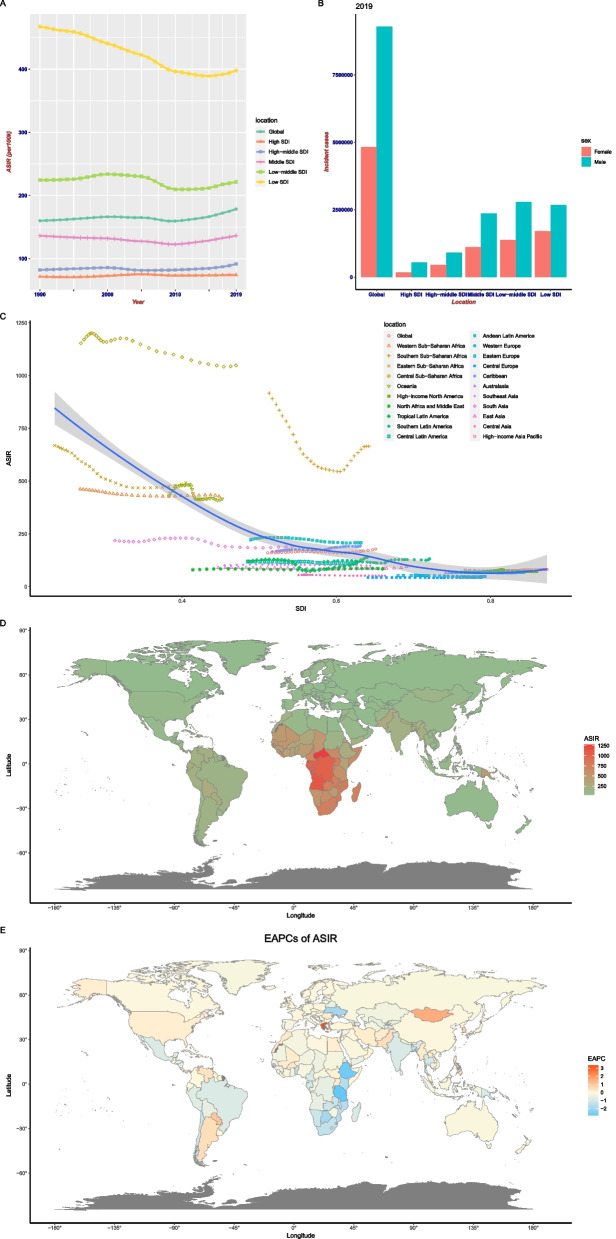
The change trends of incident cases, ASIR and EAPC among different SDI quintiles. Note: **A** indicates the variation trend of ASIR in different SDI over time, **B** indicates the incident cases of different SDI, **C** indicates the correlation between SDI and ASR among 21 regions, **D** indicates the ASIR in map, **E** indicates EAPC of ASIR in map

The global incidence of syphilis increased from 8,845,220 (95% UI: 6,562,510–11,588,860) in 1990 to 14,114,110 (95% UI: 10,648,490–18,415,970) in 2019 (Table 1) at an approximate rate of 60%. The ASIR increased from 160.03/100,000 persons (95% UI: 120.66–208.1) to 178.48/100,000 persons (95% UI: 134.94–232.34). The global ASIR continued to increase rapidly, especially from 2010 to 2019. In five SDI subregions, the ASIR was inversely correlated with the SDI from 1990 to 2019 and almost consistently five times higher in territories with low SDIs than in those with high SDIs (Fig. 2A). The EAPCs for middle (-0.18, 95% CI: -0.30– -0.06), low-middle (-0.27, 95% CI: -0.39 – -0.15) and low (-0.74, 95% CI: -0.80– -0.67) SDI regions showed a decreasing ASIR from 1990 to 2019. In contrast, the EAPCs for high SDI regions were greater than 0, indicating that the ASIRs were increasing (Table 1). The comprehensive ASIR chart in Fig. 2A shows that the ASIRs in low SDI regions increased annually from 2015 to 2019, and the ASIRs in other regions increased from 2010 to 2019.

**Table 1:** The incident cases and ASIR of syphilis in 1990 and 2019, and its temporal trends from 1990 to 2019

	**1990**		**2019**		**1990–2019 EAPC** **No.(95%CI)**
	**Incident cases** No.*10^**3**^**(95% UI)**	**ASIR per 100,000** **No.(95% UI)**	**Incident cases** No.*10^**3**^**(95% UI)**	**ASIR per 100,000** **No.(95% UI)**	
**Overall**	8845.22(6562.51, 11588.86)	160.03(120.66, 208.1)	14114.11(10648.49, 18415.97)	178.48(134.94, 232.34)	0.16(0.07, 0.26)
**Sex**					
Female	3355446.35(2502207.45, 4388063.8)	119.95(90.17, 156.34)	4824855.31(3725828.82, 6182484.11)	124.98(96.68, 160.25)	-0.3(-0.49, -0.11)
Male	5489771.19(4066355.47, 7208417.29)	199.14(149.81, 260.75)	9289253.08(6919196.33, 12282791.68)	231.31(171.88, 305.3)	0.43(0.38, 0.47)
**Socio-demographic index**					
High SDI	623.3(471.9, 826.55)	71.52(54.07, 94.72)	721.92(548.59, 951.88)	74.05(55.61, 98.92)	0.16(0.1, 0.22)
High-middle SDI	1011.91(754.28, 1333.58)	82.22(61.68, 108.01)	1356.68(1033.5, 1786.05)	91.55(69.25, 119.03)	0.11(0.01, 0.21)
Middle SDI	2477.83(1809.89, 3280.76)	136.26(102.1, 179.16)	3472.41(2596.1, 4596.48)	136.36(101.06, 180.12)	-0.18(-0.3, -0.06)
Low-middle SDI	2459.57(1804.25, 3253.48)	224.35(166.26, 294.69)	4169.24(3092.78, 5465.2)	221.41(165.71, 289.75)	-0.27(-0.39, -0.15)
Low SDI	2267.02(1720.58, 2889.82)	467.4(356.95, 593.73)	4384.62(3345.66, 5637.48)	398.05(306.64, 507.71)	-0.74(-0.8, -0.67)
**Region**					
Andean Latin America	83.04(61.3, 110.51)	221.73(167.05, 293.67)	138.14(104.7, 180.53)	207.84(158.51, 270.68)	-0.45(-0.54, -0.36)
Australasia	14.1(10.46, 18.96)	65.58(48.71, 87.54)	17.71(13.32, 23.46)	64.07(47.75, 85.84)	-0.12(-0.13, -0.1)
Caribbean	61.44(45.7, 81.46)	165.87(125.65, 216.09)	92.37(75.16, 113.65)	191.99(156.21, 235.61)	0.49(0.43, 0.55)
Central Asia	37.39(28.05, 49.21)	54.85(41.88, 71.72)	51.63(39.5, 66.88)	52.1(40.26, 67.34)	-0.36(-0.42, -0.3)
Central Europe	54.15(41.35, 71.11)	43.38(32.95, 56.96)	47.32(36.32, 61.63)	43.28(32.96, 56.54)	-0.02(-0.04, 0.01)
Central Latin America	195.78(143.3, 259.92)	118.92(89.05, 156.63)	296.13(227.21, 384.26)	111.77(86.08, 145.02)	-0.27(-0.33, -0.22)
Central Sub-Saharan Africa	580.89(435.01, 749.57)	1153.28(880.53, 1482.29)	1332.45(1017.53, 1723.69)	1048.4(803.52, 1344.82)	-0.5(-0.58, -0.41)
East Asia	1224.26(895.45, 1631.22)	89.28(66.65, 118.27)	1459.54(1092.02, 1958.96)	93.43(69.27, 123.92)	-0.08(-0.21, 0.04)
Eastern Europe	125.74(96.17, 165.15)	53.1(40.9, 68.98)	102.35(78.85, 136.35)	47.97(37.05, 62.56)	-0.56(-0.65, -0.48)
Eastern Sub-Saharan Africa	1181.6(916.25, 1480.69)	669.08(522.83, 834.07)	1992.07(1551.25, 2495.06)	492.63(386.33, 619.27)	-1.42(-1.61, -1.23)
High-income Asia Pacific	145.87(110.95, 192.31)	78.31(59.18, 103.74)	136.11(103.15, 181.01)	81.36(60.92, 109.14)	0.28(0.22, 0.34)
High-income North America	205.18(154.8, 273.09)	68.27(51.74, 89.69)	252.31(192.72, 331.6)	71.76(54.06, 95.06)	0.18(-0.03, 0.39)
North Africa and Middle East	262.02(189.03, 348.87)	79.67(59.21, 106.06)	562.03(415.47, 750.43)	84.2(62.65, 111.7)	0.15(0.06, 0.24)
Oceania	28.67(20.98, 37.65)	430.43(320.8, 560.81)	57.91(42.04, 76.97)	415.8(301.62, 551.31)	-0.56(-0.73, -0.38)
South Asia	2332.44(1691.29, 3098.81)	219.04(161.13, 289.42)	3725.19(2730.12, 4965.9)	190.99(140.95, 254.13)	-0.71(-0.95, -0.47)
Southeast Asia	487.64(352.37, 649.91)	101.17(74.45, 134.79)	722.67(535.24, 961.37)	99.61(73.45, 131.74)	-0.14(-0.19, -0.1)
Southern Latin America	58.65(43.84, 77.44)	117.88(88.25, 155.84)	88.79(71.68, 110.38)	131.89(106.8, 163.69)	0.26(0.08, 0.44)
Southern Sub-Saharan Africa	502.74(381.66, 648.48)	916.8(697.82, 1174.15)	571.16(420.69, 751.87)	665.35(496.08, 872.21)	-1.19(-1.69, -0.69)
Tropical Latin America	190.64(139.63, 255.94)	119.04(88.38, 158.58)	332.79(259.02, 415.93)	139.68(109.18, 174.26)	-0.66(-1.5, 0.17)
Western Europe	269.53(201.47, 355.93)	68.34(51.17, 90.38)	263(199.22, 348.2)	67.63(50.12, 90.19)	-0.03(-0.04, -0.02)
Western Sub-Saharan Africa	803.45(591.41, 1043.07)	461.93(345.94, 598.09)	1872.43(1377.02, 2458.53)	427.97(318.21, 556.32)	-0.26(-0.31, -0.2)

Note: age-standardized incidence rate (*ASIR*), estimated annual percentage change (*EAPC*), 95% uncertainty interval (*UI*), 95% confidence interval (*CI*), social development index (*SDI*)

*Reflected a number multiplied by 1,000

The global ASDR decreased from 1.55/100,000 persons (95% UI: 0.54–3.24) in 1990 to 1.28/100,000 persons (95% UI: 0.45–2.62) in 2019 (Table 2), and the age-standardized DALY rate also decreased from 135.08/100,000 persons (95% UI: 45.61–284.1) in 1990 to 113.09/100,000 persons (95% UI: 39.49–231.38) in 2019 (Table 3). Their EAPCs were -1.06 (95% CI: -0.89– -1.23) and -1.01 (95% CI: -1.18– -0.84), respectively. However, from the perspective of trend changes, the global ASDR and age-standardized DALY rate decreased rapidly before 2010 before slowing down from 2010 to 2017; there were minimal increments after 2017. Compared to the ASIR, the ASDR and age-standardized DALY rate were also inversely correlated with the SDI, and the ASDR and age-standardized DALY rate for low SDI regions were almost 100 times higher than those for high SDI regions in 1990 and 2019 (Supplementary Figs. 1A and 2A).

**Table 2:** The death cases and ASDR of syphilis in 1990 and 2019, and its temporal trends from 1990 to 2019

	**1990**		**2019**		**1990–2019** **EAPC No.(95%CI)**
	**Death cases** **No.(95% UI)**	**ASDR per 100,000** **No.(95% UI)**	**Death cases** No.*(95% UI)	**ASDR per 100,000** **No.(95% UI)**	
**Overall**	100450.82(34478.61, 210456.18)	1.55(0.54, 3.24)	83681.79(29799.16, 170424.22)	1.28(0.45, 2.62)	-1.06(-0.89, -1.23)
**Sex**					
Female	45227.69(15501.8, 95529.22)	1.43(0.5, 3.02)	37457.51(13274.55, 76846.46)	1.19(0.42, 2.45)	-1.05(-1.23, -0.87)
Male	55223.13(19228.79, 116468.77)	1.68(0.61, 3.49)	46224.28(16529.86, 94506.42)	1.37(0.49, 2.81)	-1.08(-1.24, -0.92)
**Socio-demographic index**					
High SDI	381.33(240.9, 624.42)	0.05(0.03, 0.1)	225.08(122.03, 389.74)	0.03(0.01, 0.07)	-0.83(-0.42, -1.25)
High-middle SDI	3057.75(1306.36, 6083.44)	0.3(0.13, 0.6)	1853.86(707.94, 3843.28)	0.23(0.08, 0.49)	-1.09(-0.91, -1.28)
Middle SDI	16775.83(5866.53, 35721.78)	0.83(0.3, 1.75)	12510.01(4257.98, 26469.89)	0.72(0.24, 1.53)	-0.59(-0.55, -0.64)
Low-middle SDI	30373.68(10158.33, 64878.54)	1.76(0.64, 3.69)	20727.25(7210.85, 43459.95)	1.23(0.43, 2.57)	-1.64(-1.39, -1.89)
Low SDI	49767.85(17165.84, 104214.4)	4.65(1.71, 9.57)	48250.01(17373.4, 98217.26)	2.74(1.01, 5.54)	-2.12(-1.98, -2.26)
**Region**					
Andean Latin America	1177.14(360.49, 2564.95)	2.08(0.66, 4.5)	576.68(195.25, 1241.51)	0.91(0.31, 1.97)	-3.92(-3.6, -4.24)
Australasia	4.36(3.47, 5.16)	0.02(0.02, 0.02)	2.46(1.82, 3.3)	0.01(0, 0.01)	-3.69(-2.52, -4.84)
Caribbean	825.89(297.71, 1726.95)	1.95(0.73, 4.02)	986.9(368.8, 2004.84)	2.5(0.92, 5.1)	0.6(0.94, 0.26)
Central Asia	146.97(78.78, 256.62)	0.19(0.11, 0.31)	123.93(44.64, 245.29)	0.14(0.05, 0.27)	-1.78(-1.34, -2.22)
Central Europe	53.16(27.03, 100.01)	0.06(0.03, 0.11)	15.91(7.01, 31.52)	0.03(0.01, 0.06)	-3.06(-2.81, -3.31)
Central Latin America	270.63(202.22, 382.23)	0.14(0.11, 0.19)	110.23(74.98, 167.18)	0.05(0.03, 0.08)	-3.68(-3.46, -3.9)
Central Sub-Saharan Africa	8915.48(3085.41, 18093.59)	7.46(2.78, 14.83)	8586.87(2975.85, 17665.19)	4.16(1.49, 8.48)	-2.67(-2.39, -2.96)
East Asia	4729.85(1663.84, 9944.49)	0.4(0.15, 0.83)	1999.23(705.64, 4395.83)	0.25(0.08, 0.57)	-0.88(-0.56, -1.19)
Eastern Europe	172.81(93.07, 193.89)	0.07(0.04, 0.08)	27.1(21.31, 50.54)	0.01(0.01, 0.02)	-9.75(-8.09, -11.37)
Eastern Sub-Saharan Africa	30239.21(10599.42, 62722.64)	7.65(2.91, 15.35)	25783.67(9524.82, 51886.89)	3.97(1.54, 7.9)	-2.56(-2.33, -2.8)
High-income Asia Pacific	92.19(49.84, 170.05)	0.08(0.03, 0.16)	53.6(24.78, 102.69)	0.06(0.02, 0.14)	-0.13(0.21, -0.46)
High-income North America	66.51(55.44, 70.78)	0.02(0.02, 0.02)	39.14(34.44, 46.15)	0.01(0.01, 0.01)	-3.52(-3.01, -4.03)
North Africa and Middle East	2650.46(815.45, 6102.54)	0.48(0.15, 1.1)	2569.65(809.02, 5604.95)	0.44(0.14, 0.96)	-1.32(-0.91, -1.73)
Oceania	626.77(210.18, 1333.79)	5.97(2.03, 12.67)	1084.37(363.94, 2200.24)	5.56(1.89, 11.25)	-0.76(-0.52, -1)
South Asia	26799.73(8383.82, 58292.62)	1.65(0.57, 3.5)	14272.31(4795.63, 30905.7)	0.9(0.31, 1.94)	-2.66(-2.12, -3.19)
Southeast Asia	5751.8(1815.41, 13013.94)	0.98(0.32, 2.18)	5639.9(1777.16, 12322.96)	1.07(0.33, 2.34)	0.47(0.68, 0.26)
Southern Latin America	85.52(74.4, 96.43)	0.17(0.15, 0.2)	27.23(21.12, 34.95)	0.05(0.04, 0.06)	-3.98(-3.36, -4.59)
Southern Sub-Saharan Africa	4496.69(1618.83, 9076.54)	6.27(2.3, 12.52)	2961.08(1044.09, 5975.18)	3.73(1.33, 7.51)	-1.81(-1.42, -2.19)
Tropical Latin America	692.42(519.77, 940.23)	0.42(0.32, 0.57)	471.78(314.05, 716.32)	0.29(0.19, 0.45)	-0.84(-0.26, -1.41)
Western Europe	171.18(126.49, 238.52)	0.05(0.03, 0.07)	75.27(40.39, 133.76)	0.03(0.01, 0.05)	-0.73(-0.05, -1.4)
Western Sub-Saharan Africa	12482.04(4037.37, 27121.98)	3.02(1.01, 6.53)	18274.47(6046.07, 38226.97)	2.36(0.79, 4.93)	-0.7(-0.51, -0.88)

Note: age-standardized death rate (*ASDR*), estimated annual percentage change (*EAPC*), 95% uncertainty interval (*UI*), 95% confidence interval (*CI*), social development index (*SDI*)

*Reflected a number multiplied by 1,000

**Table 3:** The DALYs and Age-standardized DALY rate of syphilis in 1990 and 2019, and its temporal trends from 1990 to 2019

	1990		2019		1990–2019 EAPC No.(95%CI)
	DALYs No.*10^3^(95% UI)	Age-standardized DALY rate per 100,000 No.(95% UI)	DALYs No.*10^3^(95% UI)	Age-standardized DALY rate per 100,000 No.(95% UI)	
Overall					-1.01(-1.18,-0.84)
Sex					
Female	3991.16(1356.98,8447.46)	126.22(43.07,266.91)	3297.86(1154.33,6788.2)	104.97(36.61,216.29)	-1.05(-1.23,-0.87)
Male	4808.67(1619.31,10225.72)	143.55(49.12,304.07)	4060.73(1426.88,8349.35)	120.71(42.23,248.61)	-0.98(-1.14,-0.82)
Socio-demographic index					
High SDI	27.43(15.12,48.82)	4.15(2.01,7.89)	20.92(11.67,35.88)	3.14(1.35,6.09)	-0.31(-0.62,0)
High-middle SDI	252.9(97.37,519.21)	25.07(9.5,51.71)	162.16(60.19,338.12)	20.47(7.11,43.56)	-0.74(-0.93,-0.56)
Middle SDI	1466.39(500.36,3142.53)	71.6(24.73,152.86)	1095.9(363.17,2336.66)	63.01(20.64,134.88)	-0.53(-0.58,-0.49)
Low-middle SDI	2663.3(863.45,5722.16)	149.97(50.18,320.24)	1813.84(604.89,3835.75)	106.78(35.61,225.77)	-1.56(-1.82,-1.3)
Low SDI	4381.52(1480.77,9208.55)	400.33(139.67,834.95)	4255.62(1525.62,8683.2)	239.21(86.63,486.2)	-2.08(-2.22,-1.94)
Region					
Andean Latin America	103.82(31.35,226.75)	181.46(55.55,395.49)	50.57(16.59,109.13)	80.07(26.29,172.88)	-3.93(-4.26,-3.6)
Australasia	0.18(0.15,0.22)	0.96(0.81,1.16)	0.09(0.07,0.12)	0.26(0.21,0.35)	-3.38(-4.18,-2.58)
Caribbean	71.77(24.6,151.55)	167.13(58.35,351.84)	85.92(31.3,176.15)	218.89(79.4,449.64)	0.64(0.31,0.98)
Central Asia	10.99(5.02,20.69)	12.81(6.38,23.21)	10.49(3.48,21.23)	11.51(3.81,23.28)	-0.61(-0.94,-0.28)
Central Europe	4.06(1.8,8.2)	4.62(1.82,9.73)	1.47(0.68,2.84)	2.39(0.89,5.04)	-2.72(-2.97,-2.47)
Central Latin America	22.53(16.51,32.3)	10.53(8.01,14.66)	8.9(5.94,13.81)	3.98(2.58,6.28)	-3.61(-3.81,-3.41)
Central Sub-Saharan Africa	785.02(268.86,1596.57)	635.77(220.17,1285.86)	757.84(263.06,1563.1)	360.36(125.48,740.47)	-2.63(-2.93,-2.34)
East Asia	405.56(133.68,867.63)	33.79(11.19,72.11)	175.05(59.78,388.92)	22.3(6.96,50.72)	-0.75(-1.07,-0.44)
Eastern Europe	7.1(4.37,7.85)	3.13(2.1,3.43)	1.52(1.26,2.28)	0.88(0.74,1.16)	-7.8(-9.32,-6.25)
Eastern Sub-Saharan Africa	2657.14(920.02,5531.37)	650.73(232.88,1341.61)	2267.48(835.68,4576.73)	342.64(128.09,687.87)	-2.52(-2.76,-2.27)
High-income Asia Pacific	6.85(3.06,13.73)	6.3(2.35,13.53)	4.84(2.34,9.08)	5.87(2.13,12.31)	0.23(-0.04,0.51)
High-income North America	5.34(4.37,6.52)	1.75(1.45,2.12)	4.88(3.8,6.2)	0.99(0.78,1.23)	-2.21(-2.45,-1.96)
North Africa and Middle East	234.41(71.52,540.53)	42.21(13.03,97.07)	226.06(69.41,494.69)	38.72(11.9,84.73)	-1.34(-1.75,-0.92)
Oceania	55.51(18.62,118.22)	526(176.48,1119.12)	96.01(32.1,194.98)	490.51(165.24,994.81)	-0.77(-1,-0.53)
South Asia	2351.69(718.32,5148.19)	139.77(43.82,303.9)	1244.5(397.9,2719.78)	77.52(24.7,169.58)	-2.57(-3.12,-2.02)
Southeast Asia	504.52(155.37,1148.08)	84.5(26.57,191.44)	495.37(155.11,1088.5)	93.88(29.32,206.71)	0.52(0.31,0.74)
Southern Latin America	6.18(5.38,7.08)	12.38(10.78,14.18)	1.92(1.44,2.51)	3.72(2.72,4.99)	-3.77(-4.35,-3.2)
Southern Sub-Saharan Africa	394.66(139.02,800.77)	543.22(195.26,1096.38)	260.17(90.53,527.49)	326.64(113.78,661.99)	-1.76(-2.16,-1.36)
Tropical Latin America	59.78(44.46,81.8)	35.41(26.4,48.32)	39.82(25.77,61.28)	24.91(15.9,38.78)	-0.8(-1.38,-0.21)
Western Europe	8.85(5.61,14.53)	2.98(1.56,5.56)	5.89(2.94,10.93)	2.3(0.88,4.67)	0.3(-0.25,0.86)
Western Sub-Saharan Africa	1103.87(355.23,2401.58)	265.1(86.97,574.72)	1619.79(535.87,3389.37)	209.12(69.38,436.87)	-0.66(-0.84,-0.47)

Note: disability adjusted life-years (*DALY*), estimated annual percentage change (*EAPC*), 95% uncertainty interval (*UI*), 95% confidence interval (*CI*), social development index (*SDI*)

*Reflected a number multiplied by 1,000

With the increase in the SDI, the changes in the ASIR, ASDR, and age-standardized DALY rate in 2019 were similar (*P* < 0.05) (Supplementary Fig. 3A, B, and C), and Pearson's coefficient was negative; however, the ASDR and age-standardized DALY rate peaked when the SDI approached 0.3. The EAPC was negatively correlated with the ASIR but positively correlated with the ASDR and age-standardized DALY rate in 1990 (Supplementary Fig. 3D, E, and F).

### Global burden of syphilis by region

In the 21 regions, South Asia recorded the highest number of incident cases at 2,332,440 (95% UI: 1,691,290–3,098,810) in 1990, and Australia recorded the lowest number of incident cases at 14,100 (95% UI: 10,460–18,960). Central Sub-Saharan Africa recorded the highest ASIR of 1,153.28/100,000 persons (95% UI: 880.53–1,482.29), while South Asia ranked seventh (Table 1). In 2019, Central Sub-Saharan Africa still recorded the highest ASIR among the 21 regions. In South Asia, the annual cases increased by 59.7% from 1990 to 2019, but the ASIR decreased to 190.99/100,000 persons (95% UI: 140.95–254.13), and the EAPC was -0.71 (95% CI: -0.95– -0.47) (Table 1). The situations in most regions were similar to those in South Asia, but European countries had fewer cases and lower ASIRs in 2019 than in 1990; given the negative EAPC, the number of incident cases and ASIR were much lower than for other territories.

**Fig. 3:**
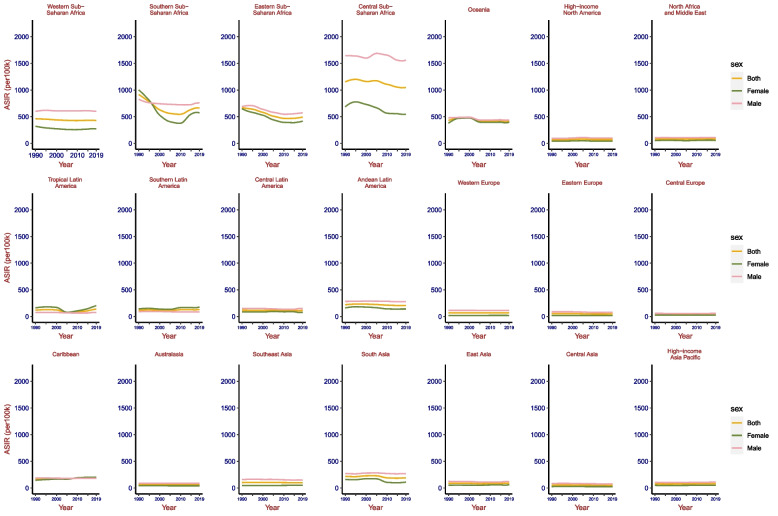
The trend of ASIR with the year among 21 regions

From 1990 to 2019, Central Sub-Saharan Africa and many other African regions consistently had the highest ASIRs among the 21 regions (Figs. 2C and 3). In Latin America, the ASIR for Andean Latin America was the highest, and that of South Asia was the highest in Asia. The ASIR was generally lower for Europe than for other regions. As shown in Fig. 3, the ASIR has shown the most rapid increase since 2005 for Tropical Latin America and has increased rapidly since 2010 after a sharp decline for Southern Sub-Saharan Africa. Although the ASIR in Central Sub-Saharan Africa fluctuated, it tended to decline overall.

Central Sub-Saharan Africa consistently had a low SDI and a high ASIR between 1990 and 2019 (Fig. 2C), while Western Sub-Saharan Africa and Eastern Sub-Saharan Africa had less than half of the ASIR of Central Sub-Saharan Africa with similar SDIs. Southern Sub-Saharan Africa had a moderate SDI, but its ASIR was markedly higher than those of other regions with similar SDIs.

The map in Fig. 2D shows darker red areas corresponding to Central and Southern Sub-Saharan Africa, indicating higher ASIRs for these regions in 2019. The EAPC for the ASIR is represented by the deepest red on the map for Greece, followed by that for Mongolia, indicating that their ASIRs were increasing at almost the fastest rate worldwide (Fig. 2E). In addition, from the base data, India and China had the highest numbers of incident cases, 2,729,026.77 (95% UI: 2,009,271.45–3,626,658.48) and 1,411,512.57 (95% UI: 1,055,475.01–1,896,966.91), respectively, among 204 countries and territories in 2019 (Supplementary Table 1).

In 1990, the three regions with the highest ASDRs were Central Sub-Saharan Africa, Eastern Sub-Saharan Africa, and Southern Sub-Saharan Africa (Table 2). The same pattern was observed for the age-standardized DALY rate (Table 3). In 2019, with the global decline in the ASDR and age-standardized DALY rate, the EAPC of the ASDR in Eastern Europe was -9.75 (95% CI: -8.09– -11.37), and the EAPC of the age-standardized DALY rate was -7.80 (95% CI: -9.32– -6.25), which was the maximum for the 21 regions. The ASDR and age-standardized DALY rate are still increasing in the Caribbean and Southeast Asia. Eastern Sub-Saharan Africa, Central Sub-Saharan Africa, and Oceania have low-level SDIs, and their ASDRs and age-standardized DALY rates are nearly the highest in the world (Supplementary Figs. 1C and 2C). Among the 204 countries and territories, the two countries with the greatest number of deaths in 2019 were Nigeria, located in Africa, and India, 8,785.56 (95% UI: 2,805.97–20,068.23) and 7,175.64 (95% UI: 2,417.34–15,408.55), respectively (Supplementary Table 2). Even though the number of incident cases in China ranked second among 204 countries and territories, after India, the number of deaths in China ranked fifteenth, substantially lower than those in India in 2019. Similarly, Nigeria and India also had the highest DALYs among these territories, 779,214.85 (95% UI: 248,649.87–1,780,486.93) and 621,529.6 (95% UI: 198,919.02–1,350,394.75), respectively (Supplementary Table 3). Additional visual conditions for the ASDR and age-standardized DALY rate are shown in Supplementary Figs. 1 and 2.

### Global burden of syphilis by age and sex

**Fig. 4:**
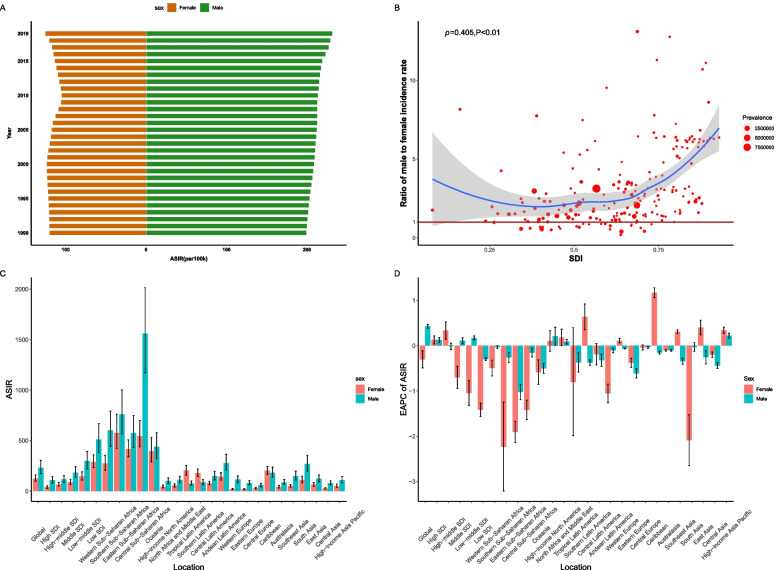
The ASIR, SDI and EAPC of male and female. Note: **A** indicates ASIR in male and female from 1990 to 2019, **B** indicates the correlation between the ration of female to male incident cases and SDI, **C** indicates the ASIR of male and female in 21 regions, **D** indicates the EAPC of ASIR in male and female among 21 regions

Intuitively, both globally and in the five SDI regions, the incident cases, deaths and DALYs among males were higher than those among females (Fig. 2B, Supplementary Figs. 1B and 2B). Similarly, the ASIR, ASDR, and age-standardized DALY rate for males have always been markedly higher than those for females (Tables 1, 2 and 3 and Fig. 4A). The ASIR of syphilis has increased worldwide but has declined for females, with an EAPC of -0.30 (95% CI: -0.49– -0.11) (Table 1 and Fig. 4D). Only Tropical Latin America and Southern Latin America showed ASIRs for females higher than those for males for the entire 30 years; in Tropical Latin America, the male ASIR has stabilized, but the female ASIR has surged rapidly in recent years. The ASIR for females declined rapidly to become lower than that for males in Southern Sub-Saharan Africa, while it increased slowly to become greater than that for males in the Caribbean (Figs. 3 and 4C). There was an overall inverse relationship between the ratio of male to female incidence rate and SDI in 204 countries and territories, and the inverse relationship is more pronounced after SDI greater than 0.5 (Fig. 4B).

**Fig. 5:**
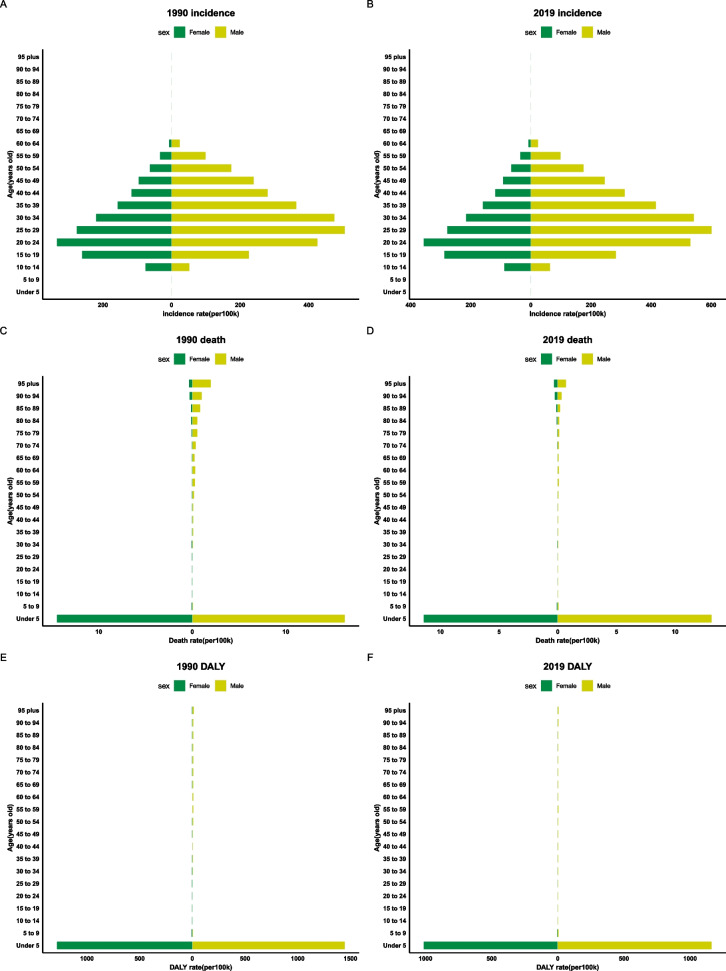
The incidence, death and DALYs rates of male and female at different ages in 1990 and 2019. Note: **A** indicates the incidence rate of male and female at different ages in 1990, **B** indicates the incidence rate of male and female at different ages in 2019, **C** indicates the death rate of male and female at different ages in 1990, **D** indicates the death rate of male and female at different ages in 2019, **E** indicates the DALYs rate of male and female at different ages in 1990, **F** indicates the DALYs rate of male and female at different ages in 2019

The ASDR and age-standardized DALY rate for both males and females have declined globally, but they were always higher for males than for females (Tables 2 and 3). In 1990 and 2019, the incidence of syphilis showed a unimodal peak for all ages for both males and females, with a peak at 20–24 years for females and 24–29 years for males. There were more infected female than male patients younger than 19 years and older than 19 years (Fig. 5A and B). Children under the age of five had a higher death rate and DALY rate than any other age group in both 1990 and 2019 (Fig. 5C, D, E, F).

## Discussion

This study statistically estimated the global syphilis situation from 1990 to 2019 using data from the GBD 2019. Overall, there was a minimal increase in the morbidity rate, whereas the mortality rate and DALY rate decreased during the past 30 years. Although the overall situation of syphilis has improved slightly, there are still large differences in terms of the syphilis situation in different regions of the world, which is closely related to the economic, medical and educational levels of different regions and countries. For example, in areas with high SDIs, syphilis transmission is relatively less widespread, while in areas with low SDIs, syphilis is more widespread. Therefore, this study evaluated the situation of syphilis at the global level and regional level from various aspects.

The GBD database divides the world into 21 unique regions. Sub-Saharan Africa has always had a higher burden of syphilis [15]; its ASIR was generally high, and the ASIRs for Central and Southern Sub-Saharan Africa were the highest. Africa has had a long history of slow and inefficient development, which has markedly been less rapid than that of other regions; this is partially attributed to the subpar medical system. The lack of a comprehensive system of prevention, screening, and treatment [16], coupled with the tropical climate in Africa, facilitates the spread of several diseases. The lack of medical treatment facilities and disease prevention strategies and the weak economy in areas in Africa facilitate the wanton spread of diseases without any obstacles. The Central African Republic, which had the highest incidence among the 204 countries and regions, unsurprisingly had a low SDI. In contrast, the ASIR for Europe was generally low. Regions such as Europe are generally considered to be well developed, and they have medium or higher SDIs, most of which are high-middle or high. However, the prevalence of syphilis has continued to increase in these high-income countries in recent years [6]. As seen in the results, the ASIR of syphilis increased worldwide, especially in two countries, Greece and Mongolia, where small populations play an important role. China and India have large populations; large numbers of incident cases were hidden in large populations. The result, therefore, was a large number of incident cases with an inconspicuous upward trend.

The GBD data showed that the ASDR and age-standardized DALY rate overlapped both globally and for the 21 individual regions. Their change patterns were similar to those of the ASIR. The ASDR and age-standardized DALY rate were higher in low-SDI areas but lower in high-SDI areas. The inadequacy of medical care leaves syphilis sufferers with little hope and a lifetime of caution. Especially in the late stages of syphilis, the consequences can be lifelong disability and even loss of life expectancy when the disease affects the cardiovascular and nervous systems. For example, TP invades arteries and causes arteritis, which may develop into more serious pathological phenomena, such as aneurysms [17]. Other complications include blindness and permanent hearing loss [18] caused by syphilis. The above adverse effects of syphilis could directly lead to an increase in YLL and YLD without prevention and control, which is ultimately reflected in the DALY burden. However, countries and regions with high ASDRs and age-standardized DALY rates are still concentrated in sub-Saharan Africa.

Before 2015, the ASIR, ASDR, and age-standardized DALY rate of syphilis had all substantially decreased, especially in areas with low and low-middle SDIs. This implies that with the development of comprehensive capabilities worldwide, there have been improvements in each region. However, the ASIR began increasing again after 2015 [19], which means that there are unpredictable difficulties in preventing or treating syphilis, leading to a resurgence in syphilis incidence, mortality and DALYs. These are global changes in the ASIR. However, the ASIR declined in most regions between 1990 and 2019.

Although treatment and prevention strategies are available, syphilis has not yet been completely eliminated. Therefore, there is still a long way to go in the fight against syphilis. With the advent of penicillin, the first kind of antibiotic, within the last century [20], syphilis is no longer incurable. Early syphilis can be cured with long-term penicillin treatment [21], but it is often difficult for patients to receive continuous penicillin treatment and titre testing. Patients often return to normal life without complete recovery or regular testing because syphilis is difficult to control. Moreover, the adverse consequence of the long-term use of antibiotics is resistance to antibiotics, such as penicillin and macrolides, which are the first- and second-line drugs for the treatment of syphilis, respectively [22]. However, antibiotic resistance due to misuse is now a global problem, not just a problem of syphilis treatment. On the other hand, screening is a pivotal step in syphilis prevention [23], and all countries and territories cannot perfectly prevent syphilis. All these factors may lead to the spread of syphilis and make it more difficult to control, so this is not a good trend for global public health prevention and control. To solve the problem of syphilis completely, it is necessary to find a new method for antibiotic treatment and prevention screening.

The high incidence rates in both males and females were concentrated in the adolescent stage. However, the peak age of incidence rate in females is 20 to 24 years, whereas in males it is 25 to 29 years. This is associated with slightly earlier exposure to sex, including precocious puberty and forced sexual assault, in females than in males [24]. The increase in the number of men who have sex with men can not only lead to wide spread of syphilis in males but also in heterosexual relationship [25]. From this perspective, reasonable and safe sexuality erotism can be helpful in the control of syphilis.

Strikingly, the ASDR and age-standardized DALY rate of syphilis among children under 5 years of age were much higher than those of any other age groups, and even played a decisive role in death and DALY burden globally. Pregnant women infected with syphilis who are not cured will transmit syphilis to the foetus, which can lead to stillbirth or neonatal death [26], or even if born alive, the children can be crippled for life by syphilis, leading to severe mortality and DALYs among children under 5 years of age. According to the World Health Organization [27], more than 500,000 cases of congenital syphilis were recorded in 2016, including more than 200,000 stillbirths and deaths. Such a high mortality rate makes congenital syphilis the second leading cause of stillbirth after malaria globally. Congenital syphilis demonstrated an increasing prevalence in several parts of developed European countries in 2011 [28]. Pregnant women who are infected with syphilis after the prenatal exam can go unnoticed and are more difficult to treat [29]. Therefore, maternal health care after pregnancy is essential for managing syphilis [30], and regular prenatal care is the best way to monitor the foetal condition.

This study had the following highlights. First, the GBD 2019 is one of the most complete databases on the global burden of disease and contains data on 354 diseases in 204 countries and territories from 1990 to 2019. Second, this study estimated the global burden of syphilis from 1990 to 2019 by SDI, sex and age in 21 regions. This will help researchers to more clearly understand the characteristics of the syphilis epidemic over the last 30 years, and at the same time, allow people to pay more attention to the target population of syphilis. Third, age-standardized rates can eliminate the influence of age and better compare the trend of syphilis between different countries and territories, supplemented by the EAPC to obtain trends over the 30-year period. Finally, in addition to the tables, this study adopted figures to visually present the research results and make more targeted statistical analyses of SDIs, sex and age in 21 regions.

However, this study has the following limitations. First, the GBD database does not have accurate records of all syphilis cases, leading to an underestimation. The database includes low-quality evidence from underdeveloped regions and outdated data. In addition, some individuals may avoid screening or hide their pathogenetic condition because of sexual shame, and some individuals cannot be detected from serological tests [31] in the early incubation period due to the absence of obvious symptoms, both resulting in the omission of the fact that syphilis is present. These above factors may have led to an underestimation of the data. Second, this study could not estimate trends in syphilis from a racial perspective because specific categories of race were not included in the GBD database. Finally, the smallest estimated units in the GBD database are countries and territories, and the data could not be further compared by province, urban or rural categories.

## Conclusion

In general, the incidence of syphilis is increasing worldwide, but the mortality rate and DALYs have improved. Although the incidence of sexually transmitted diseases has decreased in Sub-Saharan Africa, it is still markedly higher than in other regions. In South Asia, the incidence is not significant; however, the number of cases is high, which places a considerable burden on public health prevention and control. The incidence of syphilis is highest among adolescents, and adolescent sex education should be given urgent attention. If appropriate steps are not taken, an increase in the incidence of syphilis will place substantial stress on humans.

## Supplementary Information

**Additional file 1:** **Supplementary Table 1.** The incident cases and ASIR of syphilis in 1990 and 2019, and its temporal trends from 1990 to 2019. **Supplementary Table 2.** The death cases and ASDR of syphilis in 1990 and 2019, and its temporal trends from 1990 to 2019. **Supplementary Table 3.** The DALYs and Age-standardized DALY rate of syphilis in 1990 and 2019, and its temporal trends from 1990 to 2019. **Supplementary Figure 1.** The change trends ofdeath cases, ASDR and EAPC among different SDI quintiles. **SupplementaryFigure 2.** The change trendsof DALYs, age−standardized DALY rate and EAPC among different SDI quintiles. **SupplementaryFigure 3.** The correlationbetween ASIR, ASDR, age−standardized DALY in 2019 and SDI, and the correlationbetween EAPCs and ASIR, ASDR, DALY in 1990.

## Data Availability

To download the data used in these analyses, please visit the Global Health Data Exchange GBD 2019 data-input sources tool at http://ghdx.healthdata.org/gbd-2019/data-input-sources. No permission is required for anyone to access this data.

## Abbreviations

ASDR: Age-standardized death rate
ASIR: Age-standardized incidence rate
CI: Confidence interval
DALY: Disability-adjusted life year
EAPC: Estimated annual percentage change
GBD: Global Burden of Disease
GHDx: Global Health Data Exchange
SDI: Socio-demographic index
TP: Treponema pallidum
