# Supplementary material for: Global, regional, and national trends of syphilis from 1990 to 2019: the 2019 global burden of disease study
Source: BMC Public Health. 2023 Apr 24;23:754. doi: 10.1186/s12889-023-15510-4 (PMC10124004; doi:10.1186/s12889-023-15510-4)
Supplement: Supplementary file 1 — Additional file 1: Supplementary Table 1. The incident cases and ASIR of syphilis in 1990 and 2019, and its temporal trends from 1990 to 2019. Supplementary Table 2. The death cases and ASDR of syphilis in 1990 and 2019, and its temporal trends from 1990 to 2019. Supplementary Table 3. The DALYs and Age-standardized DALY rate of syphilis in 1990 and 2019, and its temporal trends from 1990 to 2019. Supplementary Figure 1. The change trends ofdeath cases, ASDR and EAPC among different SDI quintiles. SupplementaryFigure 2. The change trendsof DALYs, age−standardized DALY rate and EAPC among different SDI quintiles. SupplementaryFigure 3. The correlationbetween ASIR, ASDR, age−standardized DALY in 2019 and SDI, and the correlationbetween EAPCs and ASIR, ASDR, DALY in 1990. [file 12889_2023_15510_MOESM1_ESM.docx]

**Supplementary Materials**

**Supplementary Table 1** The incident cases and ASIR of syphilis in 1990 and 2019, and its temporal trends from 1990 to 2019

**Supplementary Table 2** The death cases and ASDR of syphilis in 1990 and 2019, and its temporal trends from 1990 to 2019

**Supplementary Table 3** The DALYs and Age-standardized DALY rate of syphilis in 1990 and 2019, and its temporal trends from 1990 to 2019

**Supplementary Figure 1** The change trends of death cases, ASDR and EAPC among different SDI quintiles

**Note:** A: Variation trend of ASDR in different SDI over time. B: Death cases of different SDI. C: The correlation between SDI and ASDR among 21 regions. D: ASDR in map. E: EAPC in map.

**Supplementary Figure 2** The change trends of DALYs, age−standardized DALY rate and EAPC among different SDI quintiles

**Note:** A: Variation trend of age−standardized DALY rate in different SDI over time. B: DALYs of different SDI. C: The correlation between SDI and age−standardized DALY rate among 21 regions. D: Age−standardized DALY rate in map. E: EAPC in map.

**Supplementary Figure 3** The correlation between ASIR, ASDR, age−standardized DALY in 2019 and SDI, and the correlation between EAPCs and ASIR, ASDR, DALY in 1990.

**Note:** A: The correlation between ASIR in 2019 and SDI. B: The correlation between ASDR in 2019 and SDI. C: The correlation between age−standardized DALY rate and SDI. D: The correlation between EAPCs and ASIR in 1990. E: The correlation between EAPCs and ASDR in 1990. F: The correlation between EAPCs and age−standardized DALY rate in 1990.

**Supplementary Table 1** The incident cases and ASIR of syphilis in 1990 and 2019, and its temporal trends from 1990 to 2019

|  | **1990** | |  | **2019** | |  | **1990-2019** |
| --- | --- | --- | --- | --- | --- | --- | --- |
|  | **Incident cases** | **ASIR per 100,000** |  | **Incident cases** | **ASIR per 100,000** |  | **EAPC** |
|  | **No.(95% UI)** | **No.(95% UI)** |  | **No.(95% UI)** | **No.(95% UI)** |  | **No.(95% CI)** |
| **Afghanistan** | 9012.74(6553.4,12104.19) | 95.1(70.08,126.09) |  | 37632.22(26798.9,50400.45) | 103.3(75.87,137.53) |  | 0.25(0.2,0.3) |
| **Albania** | 1355.68(1019.23,1789.62) | 40.1(30.59,52.45) |  | 1075.14(821.69,1410.3) | 39.12(29.65,52.02) |  | -0.1(-0.14,-0.06) |
| **Algeria** | 20292.31(14659.55,27142.2) | 85.05(63.24,113.24) |  | 36445.44(27096.64,48408.69) | 80.44(60.14,107.08) |  | -0.24(-0.39,-0.1) |
| **American Samoa** | 153.22(111.28,205.52) | 305.73(226.7,408) |  | 169.17(125.83,225.7) | 305.4(226,402.22) |  | -0.03(-0.04,-0.01) |
| **Andorra** | 46.25(33.92,61.88) | 70.27(52.27,93.58) |  | 54.82(41.84,72.38) | 67.71(50.17,90.19) |  | -0.16(-0.18,-0.13) |
| **Angola** | 117220.07(87593.98,153328.05) | 1230.46(933.24,1593.06) |  | 307093.72(227631.6,405074.67) | 1100.14(821.74,1451.68) |  | -0.5(-0.55,-0.45) |
| **Antigua and Barbuda** | 84.54(61.85,114.03) | 134.12(100.65,177.76) |  | 128.93(97.74,168.02) | 133.89(100.93,175.64) |  | -0.04(-0.09,0.02) |
| **Argentina** | 42108.33(31375.17,55347.6) | 130.58(97.1,171.59) |  | 70779.03(57278.86,88267.86) | 154.39(125.58,191.78) |  | 0.54(0.23,0.86) |
| **Armenia** | 1797.25(1359.81,2314.44) | 50.9(38.93,65.55) |  | 1397.25(1058.3,1848.8) | 44.97(34.08,59.58) |  | -0.7(-0.8,-0.61) |
| **Australia** | 11626.25(8615.56,15656.95) | 65.01(48.42,87.06) |  | 14987.25(11289.44,19868.3) | 63.62(47.49,85.1) |  | -0.11(-0.13,-0.1) |
| **Austria** | 5491.33(4040.92,7370.03) | 67.11(49.88,89.89) |  | 5496.92(4083.31,7352.13) | 66.22(48.24,89.03) |  | -0.08(-0.1,-0.05) |
| **Azerbaijan** | 4280.59(3179.73,5647.23) | 56.57(43.01,73.98) |  | 5925.59(4506.59,7832.48) | 51.25(39.22,67.69) |  | -0.5(-0.58,-0.42) |
| **Bahamas** | 509.43(368.31,681.74) | 175.63(129.99,231.29) |  | 739.27(572.95,934.11) | 180.8(140.3,229.8) |  | 0.4(0.29,0.5) |
| **Bahrain** | 608.81(427.34,833.57) | 96.67(71.4,130.12) |  | 1740.3(1283.73,2341.17) | 95.56(71.54,127.39) |  | 0.08(0.03,0.13) |
| **Bangladesh** | 257418.29(185748.1,340374.43) | 252.7(188.67,331.7) |  | 370366.56(275656.47,493422.52) | 213.44(160.21,283.87) |  | -1.13(-1.33,-0.94) |
| **Barbados** | 305.38(227.65,403.37) | 113.65(86.32,148.32) |  | 327.35(253.65,422.57) | 112.43(85.6,147.68) |  | -0.04(-0.06,-0.03) |
| **Belarus** | 4823.22(3723.93,6243.69) | 44.46(34.34,57.71) |  | 3976.83(3060.57,5194.47) | 40.97(31.55,52.64) |  | -0.44(-0.51,-0.38) |
| **Belgium** | 6857.57(5066.7,9208.28) | 67.39(49.42,90.36) |  | 6745.23(5077.31,9018.55) | 65.82(48.9,88.09) |  | -0.13(-0.15,-0.12) |
| **Belize** | 229.16(169.21,308.32) | 132.25(100.13,174.35) |  | 586.08(433.12,780.34) | 130.45(98.62,172.68) |  | -0.15(-0.2,-0.1) |
| **Benin** | 10542.05(7727.42,13820.65) | 254.39(190.7,330.02) |  | 27308.4(19961.67,36603.99) | 231.72(173.74,307.84) |  | -1.11(-1.47,-0.74) |
| **Bermuda** | 99.94(74.37,132.16) | 149.45(113.3,196.59) |  | 85.13(65.66,110.93) | 149.53(112.69,195.63) |  | 0.05(0.02,0.08) |
| **Bhutan** | 1938.9(1386.75,2602.79) | 303.28(221.76,398.51) |  | 2651.86(1916.02,3549.68) | 299.74(219.22,397.51) |  | -0.47(-0.64,-0.3) |
| **Bolivia (Plurinational State of)** | 15492.26(11349.81,21101.28) | 255.28(189.85,338.75) |  | 30201.3(23396.38,38706.35) | 245.51(192.64,314.14) |  | -0.52(-0.65,-0.4) |
| **Bosnia and Herzegovina** | 1948.12(1477.42,2562.4) | 39.01(29.62,51.16) |  | 1260.72(966.77,1657.86) | 38.9(29.64,51.74) |  | 0.03(0,0.05) |
| **Botswana** | 11030.68(8606.85,13545.98) | 835.96(649.73,1030.53) |  | 15556.01(11268.88,21064.04) | 586.19(427.66,779.65) |  | -1.89(-2.42,-1.36) |
| **Brazil** | 182995.43(134196.56,245901.09) | 117.15(87.05,156.31) |  | 315944.76(245610.32,397014.15) | 136.77(106.75,171.04) |  | -0.81(-1.69,0.08) |
| **Brunei Darussalam** | 224.02(163.57,303.8) | 77.17(57.71,102.61) |  | 404.66(301.58,541.36) | 75.59(56.78,100.85) |  | -0.02(-0.06,0.03) |
| **Bulgaria** | 3136.2(2402.5,4142.01) | 36.24(27.52,47.64) |  | 2328.24(1796.8,3045.04) | 36.07(27.75,47.24) |  | -0.03(-0.05,-0.01) |
| **Burkina Faso** | 26137.33(19212.97,34486.37) | 333.26(248.03,439.26) |  | 71083.76(55275.94,90128.42) | 340.63(264.25,430.58) |  | 0.35(0.24,0.46) |
| **Burundi** | 20717.7(15424.97,27002.98) | 426.92(324.83,546.67) |  | 38261.51(28523.33,49942.06) | 359.61(274.65,468.28) |  | -0.85(-1.14,-0.56) |
| **Cabo Verde** | 1634.71(1167.15,2198.16) | 518.99(381.94,687.21) |  | 3504.21(2545.75,4667.5) | 555.54(413.98,734.41) |  | 0.17(0.1,0.24) |
| **Cambodia** | 3940.02(2880.68,5283.71) | 42.06(31.08,55.81) |  | 7560.11(5547.61,10112.95) | 41.91(31.29,55.8) |  | -0.29(-0.42,-0.15) |
| **Cameroon** | 76776.23(57594.97,100294.71) | 785.78(590.37,1022.57) |  | 186334.01(133683.02,250238.12) | 628.82(457.38,832.52) |  | -0.84(-0.95,-0.74) |
| **Canada** | 15541.75(11605.19,20378.14) | 51.88(38.93,67.69) |  | 17766.08(13608.76,23274.77) | 51.47(38.57,68.45) |  | -0.09(-0.2,0.02) |
| **Central African Republic** | 31379.35(22967.38,40794.48) | 1227.03(921.46,1586.78) |  | 66910.21(50135.61,86117.32) | 1264.81(978.77,1613.28) |  | -0.22(-0.3,-0.14) |
| **Chad** | 26053.07(19050.57,34184.09) | 499.06(368.1,653.57) |  | 67928.69(49475.98,90893.58) | 475.46(354.01,630.82) |  | -0.39(-0.52,-0.27) |
| **Chile** | 12671.11(9291.05,17050.68) | 88.01(65.96,117.23) |  | 14149.95(10964.4,18673.36) | 75.8(59,100.47) |  | -1.13(-1.48,-0.78) |
| **China** | 1183220.42(864085.1,1577075.16) | 89.23(66.57,118.21) |  | 1411512.57(1055475.01,1896966.91) | 93.44(69.26,124.09) |  | -0.09(-0.22,0.04) |
| **Colombia** | 64792.83(47214.1,86264.96) | 189.71(141.47,248.12) |  | 94041.09(73005.89,121230.76) | 185.95(144.45,239.93) |  | 0.01(-0.03,0.04) |
| **Comoros** | 2471.94(1833.42,3206.93) | 581.57(442.19,741.3) |  | 3347.36(2524.68,4342.95) | 449.28(342.43,582.51) |  | -1.22(-1.39,-1.06) |
| **Congo** | 24277.8(18060.27,32303.77) | 1076.27(807.83,1395.99) |  | 55119.49(42553.95,69413.47) | 1026.97(790.87,1292.81) |  | -0.22(-0.25,-0.18) |
| **Cook Islands** | 58.2(42.67,77.48) | 300.94(223.28,397.82) |  | 49.01(36.59,64.46) | 296.94(217.94,394.89) |  | -0.07(-0.09,-0.04) |
| **Costa Rica** | 3590.52(2631.58,4811.53) | 115.56(87.36,152.74) |  | 5840.69(4386.53,7719.63) | 115.09(86.43,152.28) |  | -0.02(-0.03,-0.01) |
| **Croatia** | 2021.5(1564.55,2607.49) | 39.54(30.33,51.29) |  | 1577.02(1212.34,2050.27) | 39.4(30.06,51.35) |  | -0.01(-0.03,0.01) |
| **Cuba** | 16482.65(12050.21,22187.86) | 134.56(100.98,178.03) |  | 14270.34(11168.97,18348.12) | 128.18(98.91,166.96) |  | -0.4(-0.63,-0.17) |
| **Cyprus** | 547.35(408.23,725.93) | 67.3(50.41,89.07) |  | 912.42(673.04,1223.24) | 65.54(48.02,87.73) |  | -0.12(-0.13,-0.11) |
| **Czechia** | 4115.1(3188.67,5384.97) | 39.45(30.38,51.67) |  | 3973.3(3038.15,5229.59) | 39.67(30.05,52.16) |  | 0.02(0.01,0.04) |
| **Côte d'Ivoire** | 49195.71(36906.65,63284.4) | 419.99(323.17,536.33) |  | 99260.8(71829.13,131106.68) | 373.96(276.5,492.66) |  | -0.23(-0.36,-0.09) |
| **Democratic People's Republic of Korea** | 19124.92(14230.89,25390.57) | 88.19(65.99,117.79) |  | 25620.66(19198.51,33547.02) | 90.45(67.52,119.03) |  | 0.07(0.06,0.08) |
| **Democratic Republic of the Congo** | 393174.13(295152.63,504550.85) | 1130.08(865.77,1450.43) |  | 865114.63(660104.65,1114710.74) | 1017.04(784.79,1299.03) |  | -0.54(-0.67,-0.4) |
| **Denmark** | 3372.57(2482.51,4502.94) | 63.37(46.52,84.58) |  | 3268.12(2497.18,4258.41) | 61.87(46.08,81.38) |  | -0.19(-0.23,-0.15) |
| **Djibouti** | 1051.62(771.57,1382.78) | 223.01(170.47,288.59) |  | 2695.15(2028.37,3544) | 211.38(161.96,277.25) |  | -0.06(-0.18,0.05) |
| **Dominica** | 158.01(115.89,212.89) | 215.16(160.17,286.73) |  | 144.73(109.39,191.65) | 207.79(155.37,276.13) |  | 0(-0.04,0.04) |
| **Dominican Republic** | 13371.38(9738.62,17916.71) | 179.04(134.21,235.23) |  | 23254.61(18164.34,29306.27) | 200.23(157.74,250.41) |  | 0.14(0.01,0.26) |
| **Ecuador** | 22542.21(16600.91,30400.39) | 225.96(170.79,298.92) |  | 41557.91(30878.85,54907.7) | 224.85(168.01,295.05) |  | -0.02(-0.04,0) |
| **Egypt** | 28625.31(21252.53,37698.92) | 53.36(40.26,70.17) |  | 55971.65(41387.79,74445.94) | 54.05(40.12,71.04) |  | 0.21(0.12,0.3) |
| **El Salvador** | 4856.74(3608.94,6441.87) | 96.62(73.04,126.78) |  | 6012.97(4443.09,7987.74) | 90.82(68.28,118.45) |  | -0.31(-0.35,-0.26) |
| **Equatorial Guinea** | 4427.67(3328.37,5745.68) | 1191.36(905.65,1521.9) |  | 19615.46(14731.71,24709.44) | 1241.57(954.5,1539.81) |  | 0.09(-0.04,0.22) |
| **Eritrea** | 13133.17(9775.94,17157.65) | 485.35(365.19,629.31) |  | 27811.88(20818.04,37007.81) | 409.5(307.34,543.34) |  | -0.67(-0.74,-0.61) |
| **Estonia** | 772.86(598.14,1005.79) | 47.98(36.99,63.11) |  | 590.42(455.29,773.26) | 46.64(35.75,61.25) |  | -0.12(-0.19,-0.05) |
| **Eswatini** | 5356.21(3805.19,7013.74) | 695.22(517.85,880.24) |  | 5861.57(4392.59,7927.33) | 476.87(363.27,633.03) |  | -1.12(-1.31,-0.94) |
| **Ethiopia** | 217575.26(154229.05,296755.96) | 455.01(328.38,614.72) |  | 241268.27(171395.84,328760.46) | 230.48(170.01,308.55) |  | -2.67(-2.97,-2.37) |
| **Fiji** | 2509.52(1841.17,3350.32) | 310.06(229.47,411.13) |  | 2918.63(2168.03,3844.96) | 309.1(229.14,406.37) |  | -0.05(-0.1,0) |
| **Finland** | 3467.08(2579.15,4639.98) | 67.3(49.75,90.06) |  | 3202.33(2402.66,4242.93) | 66.52(48.7,89.15) |  | -0.05(-0.06,-0.03) |
| **France** | 39198.52(29337.28,52904.62) | 66.54(49.7,89.88) |  | 37601.86(28226.88,48701.91) | 65.46(48.04,86.05) |  | -0.09(-0.11,-0.07) |
| **Gabon** | 10406.43(7881.01,13406.2) | 1156.94(894.51,1481.55) |  | 18592.04(13727.87,24170.78) | 997.35(744.01,1290.4) |  | -0.97(-1.13,-0.81) |
| **Gambia** | 3485.98(2516.87,4605.15) | 370.83(278.29,484.34) |  | 8296.27(5971.42,11202.82) | 356.74(264.63,472.28) |  | -0.4(-0.52,-0.27) |
| **Georgia** | 3215.5(2435.77,4205.25) | 57.12(43.15,75.45) |  | 1893.12(1478.44,2416.2) | 55.06(42.9,70.63) |  | -0.62(-0.81,-0.44) |
| **Germany** | 54755.44(40708.71,71757.93) | 65.59(48.81,86.43) |  | 48707.24(36814.35,65509.49) | 64.73(47.9,87.94) |  | -0.12(-0.15,-0.09) |
| **Ghana** | 58583.07(42710.05,77114.85) | 418.31(312.64,549.01) |  | 133750.18(102785.14,170189.29) | 395.38(305.77,504.74) |  | -0.29(-0.34,-0.24) |
| **Greece** | 4476.69(3843.65,5175.87) | 43.58(37.27,50.45) |  | 5923.33(4467.4,7754.62) | 66.64(49.01,88.32) |  | 3.38(2.53,4.23) |
| **Greenland** | 40.63(29.97,54.91) | 60.06(45.24,80.05) |  | 33.21(25.14,43.2) | 57.04(42.92,74.54) |  | -0.17(-0.18,-0.15) |
| **Grenada** | 162.61(118.74,218.49) | 198.2(148.08,264.15) |  | 232.47(176.65,300.99) | 208.7(159.66,269.7) |  | 0.32(0.24,0.4) |
| **Guam** | 493.4(355.14,664.58) | 311.52(229.47,413.43) |  | 513.62(383.5,680.11) | 311.32(231.47,416.33) |  | -0.02(-0.05,0.01) |
| **Guatemala** | 7655.33(5714.28,10071.67) | 108.97(82.73,142.16) |  | 20334.65(15082.95,26671.46) | 105.49(80.53,137.87) |  | -0.28(-0.33,-0.22) |
| **Guinea** | 24146.82(17638.67,31207.36) | 447.22(333.64,574.11) |  | 48111.63(36088.16,62502.02) | 408.14(309.71,529.1) |  | -0.46(-0.61,-0.31) |
| **Guinea-Bissau** | 5610.5(4068.94,7382.01) | 592.04(435.22,760.01) |  | 9319.94(6812.44,12649.61) | 474.29(349.55,634.6) |  | -1.13(-1.52,-0.74) |
| **Guyana** | 1287.87(936.52,1703.69) | 158.11(118.51,206.3) |  | 1300.39(979.39,1699) | 154.38(117.46,200.76) |  | -0.08(-0.12,-0.04) |
| **Haiti** | 14044.75(10295.53,18318.14) | 229.94(173.08,297.05) |  | 34649.09(27933.54,42431.36) | 256.53(206.97,312.83) |  | 0.39(0.17,0.61) |
| **Honduras** | 4060.69(2996.36,5377.23) | 98.79(74.84,129.9) |  | 9392.75(7109.07,12467.81) | 91.02(69.95,119.83) |  | -0.48(-0.57,-0.39) |
| **Hungary** | 4188.45(3237,5448.37) | 40.11(30.8,52.45) |  | 3724.88(2833.82,4856.04) | 40.16(30.77,52.22) |  | 0.02(0.01,0.02) |
| **Iceland** | 182.22(134.17,242.19) | 68.06(50.55,90.54) |  | 224.32(168.42,296.92) | 66.99(49.96,89.04) |  | -0.08(-0.1,-0.06) |
| **India** | 1792136.43(1299513.14,2379412.63) | 212.13(155.34,280.75) |  | 2729026.77(2009271.45,3626658.48) | 179.56(132.72,237.45) |  | -0.86(-1.16,-0.57) |
| **Indonesia** | 169850.75(122899.58,226073.79) | 89.27(65.83,118.61) |  | 242157.45(179058.75,326250.3) | 85.37(62.83,114.79) |  | -0.2(-0.22,-0.18) |
| **Iran (Islamic Republic of)** | 22787.58(16103.26,31198.04) | 43.3(31.57,58.72) |  | 44319.24(32517.13,61430.9) | 45.9(33.72,61.88) |  | 0.21(0.08,0.35) |
| **Iraq** | 12943.57(9427.95,17434.13) | 83.46(62,111.02) |  | 38295.91(27746.97,50809.19) | 82.57(61.04,108.9) |  | -0.06(-0.08,-0.05) |
| **Ireland** | 2341.69(1737.11,3118.92) | 66(48.94,88.07) |  | 3000.62(2241.24,4031.14) | 65.11(47.88,87.66) |  | -0.09(-0.11,-0.06) |
| **Israel** | 3158.97(2307.61,4240.16) | 65(47.92,86.44) |  | 5687.28(4236.08,7626.04) | 65.21(48.13,88.1) |  | -0.02(-0.04,-0.01) |
| **Italy** | 42792.62(31684.33,56455.15) | 73.12(54.11,96.57) |  | 36037.83(27515.08,47374.82) | 70.49(52.14,92.97) |  | -0.21(-0.24,-0.18) |
| **Jamaica** | 4408.36(3184.57,5910.9) | 182.38(135.54,240.93) |  | 5559.25(4278.54,7009.35) | 179.42(139.74,224.69) |  | 0.49(0.32,0.66) |
| **Japan** | 105989.89(80630.96,140931.13) | 81.68(61.15,109.39) |  | 86800.61(65867.25,114761.99) | 81.83(61.32,109.33) |  | 0.03(-0.01,0.07) |
| **Jordan** | 3654.12(2603.1,5006.75) | 101.46(74.67,134.95) |  | 12870.23(9462.05,17137.84) | 101.68(75.52,134.61) |  | -0.02(-0.04,0) |
| **Kazakhstan** | 8647.38(6485.06,11504.88) | 51.27(39.07,67.41) |  | 9208.13(7103.09,11929.46) | 48.52(37.47,62.57) |  | -0.29(-0.37,-0.21) |
| **Kenya** | 106894.43(78132.07,142354.91) | 510.66(379.39,673.1) |  | 191609.76(140935.53,253599.43) | 374.83(282.67,492.33) |  | -1.6(-1.96,-1.24) |
| **Kiribati** | 274.22(197.22,362.1) | 357.29(262.98,468.05) |  | 459.92(346.84,589.49) | 367.36(278.2,468.8) |  | 0.11(0.06,0.16) |
| **Kuwait** | 1971.3(1407.04,2688.45) | 91.16(67.49,121.28) |  | 4874.11(3577.92,6635.56) | 84.24(62.24,111.54) |  | -0.29(-0.37,-0.21) |
| **Kyrgyzstan** | 1996.56(1491.83,2656.56) | 46.91(35.55,61.78) |  | 2901.44(2201.65,3832.75) | 42.59(32.66,56) |  | -0.56(-0.68,-0.43) |
| **Lao People's Democratic Republic** | 2846.19(2086.35,3821.38) | 75.6(56.06,101.36) |  | 6100.47(4427.42,8188.87) | 77.19(57.08,103.06) |  | 0.13(0.12,0.15) |
| **Latvia** | 1358.53(1065.65,1731.07) | 49.25(38.49,63.04) |  | 837.64(647.83,1096.11) | 45.99(35.56,59.97) |  | -0.36(-0.44,-0.29) |
| **Lebanon** | 2550.08(1853.48,3429.41) | 83.84(61.3,112.72) |  | 4619.47(3386.2,6150.68) | 84.54(62.36,113.05) |  | 0.01(-0.01,0.03) |
| **Lesotho** | 8553.85(6277.86,11423.07) | 488.89(368,630.76) |  | 9054.12(6710.66,12314.3) | 387.46(291.29,521.42) |  | -0.63(-0.81,-0.45) |
| **Liberia** | 10912.16(8155.42,14535.5) | 640.21(477.03,851.89) |  | 34098.1(25954.43,43122.8) | 650.19(500.71,822.06) |  | 0.12(-0.02,0.25) |
| **Libya** | 3367.05(2428.04,4515.88) | 87.51(64.18,115.95) |  | 6829.1(5092.94,9130.76) | 84.35(62.82,111.89) |  | -0.09(-0.12,-0.06) |
| **Lithuania** | 1765.35(1354.75,2293.15) | 46.15(35.62,60.41) |  | 1222.26(957.43,1573.08) | 44.9(35.13,57.54) |  | -0.14(-0.17,-0.11) |
| **Luxembourg** | 286.39(209.98,381.9) | 70.16(52.06,93.68) |  | 417.34(314.04,556.07) | 66.95(49.91,89.2) |  | -0.23(-0.26,-0.19) |
| **Madagascar** | 90286.01(67836.73,116237.92) | 809.63(622.47,1026.49) |  | 194731.31(154228.16,241741.46) | 717.43(574.29,895.14) |  | -0.88(-1.06,-0.7) |
| **Malawi** | 57161.87(46397.88,69117.54) | 646.69(533.85,782.47) |  | 105884.43(81748.39,135302.21) | 592.24(461.41,755.16) |  | -0.82(-1.06,-0.58) |
| **Malaysia** | 12728.55(9256.46,17169.21) | 69.91(51.42,93.57) |  | 24878.94(18112.91,33364.6) | 69.67(51.56,92.81) |  | -0.07(-0.12,-0.02) |
| **Maldives** | 142.3(104.21,190.78) | 74.32(55.64,99.04) |  | 582.07(409.7,824.19) | 85.37(62.76,114.95) |  | 0.46(0.3,0.63) |
| **Mali** | 36804.66(27015.07,47651.29) | 489.49(362.31,630.6) |  | 101089.31(74291.23,131131.3) | 495.85(371.41,635.47) |  | 0.26(0.18,0.33) |
| **Malta** | 246.63(184.54,330.65) | 64.99(48.2,86.98) |  | 263.04(196.73,352.83) | 65.56(48.7,88.05) |  | -0.01(-0.02,0.01) |
| **Marshall Islands** | 124.26(90.14,167.04) | 291.93(217.3,389.06) |  | 189.39(141.19,249.93) | 309.28(232.03,407.96) |  | 0.42(-0.06,0.9) |
| **Mauritania** | 9875.37(7254.63,12946.94) | 519.76(379.93,679.84) |  | 18886.95(13797.53,25148.01) | 472.46(349.68,616.87) |  | -0.29(-0.36,-0.22) |
| **Mauritius** | 1197.98(864.21,1641.32) | 95.68(70.67,129.15) |  | 1356.53(1013.15,1777.12) | 104.79(77.14,138.22) |  | 0.41(0.32,0.5) |
| **Mexico** | 70796.3(51816.07,94383.66) | 82.94(62.14,110.12) |  | 94665.25(71393.75,125484.73) | 71.27(53.86,94.54) |  | -0.83(-1.02,-0.64) |
| **Micronesia (Federated States of)** | 350.89(255.43,468.71) | 353.74(259.62,469.53) |  | 413.13(311.45,534.02) | 373.75(282.16,488.38) |  | 0.41(0.3,0.51) |
| **Monaco** | 18.2(13.46,23.82) | 65.81(48.22,87.95) |  | 19.27(14.79,25.5) | 65.84(48.83,88.59) |  | -0.07(-0.09,-0.05) |
| **Mongolia** | 1894.42(1379.1,2560.83) | 86.91(64.91,115.77) |  | 4080.65(3272.29,5048.28) | 124.71(100.26,153.7) |  | 1.55(1.27,1.84) |
| **Montenegro** | 261.56(197.11,344.54) | 39.93(30.27,52.63) |  | 247.12(188.5,320.85) | 39.82(30.45,51.9) |  | -0.02(-0.04,0) |
| **Morocco** | 47244.59(33654.46,62815.05) | 183.82(132.44,242.11) |  | 69801.18(50804.9,92261.2) | 181.17(131.36,239.01) |  | 0.31(-0.06,0.69) |
| **Mozambique** | 148494.56(116297.21,187020.83) | 1201.03(942.88,1524.64) |  | 214339.67(164027.01,268386.93) | 779.2(600.81,969.32) |  | -1.55(-1.72,-1.38) |
| **Myanmar** | 85184.23(60780.77,114752.56) | 203.26(144.96,270.78) |  | 121610.1(89937.83,159012.62) | 209.69(155.25,274.47) |  | 0.26(0.05,0.46) |
| **Namibia** | 7291.73(5400.54,9508.36) | 541.23(409.03,695.62) |  | 12484.22(9249.6,16898.11) | 489.73(366.1,659.31) |  | -1.07(-1.41,-0.73) |
| **Nauru** | 30.82(22.68,41.53) | 304.88(225.98,405.07) |  | 34.81(25.06,46.65) | 304.85(223.23,404.66) |  | -0.01(-0.03,0) |
| **Nepal** | 43526.83(31845.1,57447.1) | 239.3(178.62,313.19) |  | 75045.83(54546.04,100814.26) | 227.53(168.51,304.29) |  | -0.24(-0.27,-0.21) |
| **Netherlands** | 10999.79(8130.82,14622.41) | 67.55(50,89.05) |  | 10195.43(7769.37,13573.94) | 66.32(49.55,89.61) |  | -0.14(-0.16,-0.11) |
| **New Zealand** | 2469.88(1816.66,3310.28) | 68.54(50.55,91.36) |  | 2722.4(2051.42,3575.21) | 67.05(49.59,89.79) |  | -0.11(-0.14,-0.08) |
| **Nicaragua** | 3582.69(2634.53,4801.38) | 102.89(78.18,135.96) |  | 6458.22(4883.32,8397.66) | 92.17(70.92,118.73) |  | -0.63(-0.82,-0.44) |
| **Niger** | 20818.12(15236.86,27470.52) | 313.59(233.61,405.41) |  | 55344.56(40223.94,73070.5) | 294.08(219.33,390.15) |  | -0.32(-0.35,-0.28) |
| **Nigeria** | 382161.09(278083.23,500817.11) | 465.28(344.41,609.32) |  | 873480.43(633449.6,1165569.02) | 430.52(319.48,565.75) |  | -0.19(-0.28,-0.1) |
| **Niue** | 6.29(4.64,8.32) | 299.9(219.76,398.62) |  | 4.68(3.5,6.17) | 301.66(222.19,401.23) |  | 0.01(-0.01,0.02) |
| **North Macedonia** | 825.07(630.49,1086.26) | 38.89(29.71,51.14) |  | 897.93(681.97,1189.97) | 39.53(30.08,52.11) |  | 0.07(0.05,0.08) |
| **Northern Mariana Islands** | 191.79(140.72,255.3) | 330.6(247.76,437.36) |  | 131.17(98.81,170.09) | 320.44(237.99,426.34) |  | -0.12(-0.14,-0.09) |
| **Norway** | 3203.14(2402.67,4284.11) | 74.03(55.45,98.62) |  | 3813.15(2878.9,5020.33) | 74.28(55.34,98.22) |  | -0.03(-0.05,-0.02) |
| **Oman** | 1681.14(1194.95,2310.3) | 85.46(62.63,116.16) |  | 5867.32(4074.52,8017.64) | 87.06(63.95,115.2) |  | 0.07(-0.1,0.25) |
| **Pakistan** | 237421.82(170903.32,321930.41) | 240.58(175.65,320.97) |  | 548102.44(390969.05,750320.12) | 245.63(178.31,331.61) |  | 0.53(0.29,0.76) |
| **Palau** | 47.69(34.69,65.02) | 274.8(202.62,370.03) |  | 52.57(39.82,69.61) | 289.44(213.93,385.69) |  | 0.16(0.1,0.22) |
| **Palestine** | 1487.53(1059.04,2014.58) | 83.15(61.38,110.6) |  | 4278.91(3076.01,5735.93) | 83.48(61.35,111.68) |  | -0.05(-0.08,-0.02) |
| **Panama** | 3430.9(2489.15,4634.91) | 137.5(102.52,183.27) |  | 6467.51(5064.78,8249.15) | 152.33(119.58,193.98) |  | 0.56(0.44,0.67) |
| **Papua New Guinea** | 20293.45(14901.33,26492.87) | 482.87(359.63,628.61) |  | 45696.78(32959.79,60886.97) | 438.48(318.35,580.46) |  | -0.9(-1.13,-0.68) |
| **Paraguay** | 7639.94(5537.36,10218.39) | 195.2(143.73,258.37) |  | 16844.68(12146.11,22680.43) | 223.07(162.72,297.26) |  | 1.04(0.77,1.32) |
| **Peru** | 45005.84(33199.12,59653.37) | 210.42(158.76,276.11) |  | 66385.21(50645.59,88351.17) | 185.65(141.98,246.08) |  | -0.71(-0.86,-0.57) |
| **Philippines** | 68864.99(49866.4,92531.35) | 108.62(80.14,144.83) |  | 139003.09(101050.89,185511.65) | 117.25(85.95,156.21) |  | 0.43(0.36,0.51) |
| **Poland** | 16499.7(12516.54,21854.82) | 42.53(32.18,55.91) |  | 15803.99(11944.24,21046.61) | 41.52(31.38,54.85) |  | -0.11(-0.15,-0.07) |
| **Portugal** | 6801.99(5014.71,9056.01) | 67.07(49.18,89.2) |  | 6028.31(4556.04,7962.73) | 64.82(47.55,86.37) |  | -0.2(-0.23,-0.17) |
| **Puerto Rico** | 5526.87(4152.95,7253.92) | 149.74(112.87,196.77) |  | 4839.1(3724.05,6387.08) | 149.14(112.24,198) |  | 0.02(-0.01,0.05) |
| **Qatar** | 772.62(549.2,1055.9) | 127.33(94.66,168.26) |  | 6215.34(4346.12,8499.79) | 133.87(97.5,178.83) |  | 0.28(0.19,0.38) |
| **Republic of Korea** | 36729.03(28095.32,48154.26) | 71.63(55.42,92.91) |  | 43964.44(33237.13,58541.04) | 81.34(61.08,109.56) |  | 0.89(0.73,1.04) |
| **Republic of Moldova** | 2507.8(1913.88,3287.72) | 55.55(42.42,72.74) |  | 2173.69(1699.79,2788.65) | 57.55(45.46,73.27) |  | 0.28(0.19,0.36) |
| **Romania** | 13325.71(10044.82,17462.07) | 56.9(42.63,74.56) |  | 10440.27(8018.14,13544.66) | 60.18(45.59,77.25) |  | 0.24(0.16,0.31) |
| **Russian Federation** | 80292.03(61408.62,105812.92) | 50.52(38.81,66.16) |  | 73579.51(56678.36,97813.66) | 49.31(38,64.25) |  | -0.13(-0.16,-0.1) |
| **Rwanda** | 32301.91(24136.74,41992.56) | 505.19(384.71,652.61) |  | 51631.67(38961.94,69328.22) | 400.58(308.41,528.7) |  | -0.86(-0.94,-0.79) |
| **Saint Kitts and Nevis** | 63.65(46.97,85.07) | 156.33(119.23,203.09) |  | 96.88(74.42,128.03) | 152.37(115.68,202.36) |  | -0.09(-0.13,-0.06) |
| **Saint Lucia** | 280.9(202.83,378.47) | 202.09(151.42,268.22) |  | 380.36(288.69,496.83) | 205.13(155.67,268.95) |  | 0.24(0.15,0.32) |
| **Saint Vincent and the Grenadines** | 185.78(136.88,245.52) | 170.61(129.35,221.05) |  | 179.28(137.35,233.97) | 154.23(116.68,203.36) |  | -0.38(-0.49,-0.28) |
| **Samoa** | 358.57(256.79,483.09) | 227.11(167.18,302.01) |  | 458.34(337.77,610.98) | 220.82(163.07,295.05) |  | -0.13(-0.16,-0.11) |
| **San Marino** | 16.13(11.89,21.5) | 66.21(49.22,87.96) |  | 19.25(14.58,25.36) | 63.42(47.01,84.63) |  | -0.17(-0.2,-0.14) |
| **Sao Tome and Principe** | 397.48(288.16,529.11) | 384.23(284.77,509.89) |  | 796.54(593.09,1040.51) | 373.5(280.85,482.96) |  | -0.14(-0.22,-0.05) |
| **Saudi Arabia** | 13182.8(9479.48,17604.11) | 82.27(60.28,108.51) |  | 39336.62(28642.87,53854.62) | 80.49(59.14,108.25) |  | -0.12(-0.14,-0.1) |
| **Senegal** | 34491.04(24878.71,45435.61) | 521.65(383.4,682.05) |  | 73844.24(52852.39,98734.05) | 496.4(362.53,657.46) |  | -0.41(-0.48,-0.33) |
| **Serbia** | 3767.66(2919.7,4872.22) | 39.22(30.01,51.62) |  | 3272.41(2502.08,4259.7) | 39.21(29.71,51.02) |  | -0.03(-0.05,-0.01) |
| **Seychelles** | 62.79(45.4,85.24) | 82.8(61.19,111.42) |  | 90.97(67.56,121.09) | 83.08(61.15,111.53) |  | 0.04(-0.01,0.08) |
| **Sierra Leone** | 12988.7(9500.08,17002.02) | 387.56(289.91,503.9) |  | 32047.92(23133.2,43375.81) | 380.76(282.76,504.2) |  | -0.03(-0.11,0.05) |
| **Singapore** | 2924(2152.56,3950.75) | 76.98(57.78,102.39) |  | 4938.71(3659.05,6729.58) | 76.83(57.73,103.32) |  | 0.02(0,0.03) |
| **Slovakia** | 1887.26(1426.64,2450.59) | 35.07(26.43,45.38) |  | 1955.01(1494.92,2592.27) | 35.55(27.3,47.08) |  | 0.02(0,0.04) |
| **Slovenia** | 821.83(627.73,1076.1) | 39.41(30.04,51.48) |  | 765.37(584.63,1009.33) | 39.73(30.11,52.9) |  | 0.03(0.02,0.04) |
| **Solomon Islands** | 1404(1030.56,1872.26) | 424.72(313.86,551.98) |  | 2802.37(2076.58,3760.35) | 412.51(307.55,552.61) |  | -0.11(-0.21,-0.01) |
| **Somalia** | 39466.11(30706.85,49659.67) | 635.47(493.71,793.3) |  | 112870.99(85742.3,145342.11) | 600.26(461.91,764.94) |  | -0.07(-0.19,0.04) |
| **South Africa** | 428839.14(326416.02,551860.68) | 1071.29(818.26,1373.64) |  | 465084.01(340691.18,612573.55) | 750.5(554.15,989.9) |  | -1.34(-1.94,-0.73) |
| **South Sudan** | 38416.25(28460.73,50510.64) | 692.32(529.91,900.63) |  | 54670.64(41274.75,71071.51) | 640.46(486.72,828.24) |  | 0.15(0.04,0.25) |
| **Spain** | 27330.74(20355.42,36314.18) | 69.08(51.37,91.7) |  | 26948.8(20506.78,35153) | 65.86(48.88,86.73) |  | -0.22(-0.24,-0.2) |
| **Sri Lanka** | 5150.08(3841.14,6834.75) | 27.95(21.12,37.03) |  | 5646.83(4250.34,7442.8) | 25.63(19.09,34.02) |  | -0.67(-1.06,-0.28) |
| **Sudan** | 24815.89(18109.46,33473.77) | 132.08(96.79,175.3) |  | 60230.36(43564.61,80521.94) | 138.94(103.26,184.02) |  | -0.22(-0.39,-0.05) |
| **Suriname** | 447.2(330.41,596.12) | 112.05(85.01,147.7) |  | 644.86(495.69,842.41) | 109.05(82.51,143.39) |  | -0.12(-0.13,-0.1) |
| **Sweden** | 5936.69(4431.01,7867.19) | 71.2(52.57,93.79) |  | 6543.91(4915.76,8678.96) | 70.87(52.5,95.07) |  | -0.06(-0.08,-0.03) |
| **Switzerland** | 4952.47(3666.06,6586.79) | 67.66(49.94,89.62) |  | 5482.91(4121.66,7300.54) | 66.81(49.5,89.42) |  | -0.06(-0.08,-0.04) |
| **Syrian Arab Republic** | 9593.32(6988.9,12826.21) | 85.06(63.07,113.51) |  | 11355.01(8510.34,15233.89) | 78.6(58.52,104.23) |  | -0.29(-0.37,-0.2) |
| **Taiwan (Province of China)** | 21918.92(16137.24,29193.33) | 95.35(71.82,126.5) |  | 22402.86(17048.92,29244.99) | 94.53(70.51,124.89) |  | -0.08(-0.1,-0.06) |
| **Tajikistan** | 2715.59(2011.93,3568.37) | 56.63(43.61,73.6) |  | 4940.51(3724.83,6492.6) | 49.6(37.85,64.53) |  | -0.75(-0.86,-0.63) |
| **Thailand** | 84577.68(61092.01,113342.3) | 130.94(95.94,174.61) |  | 86969.11(65076.43,114251.31) | 122.2(89.87,161.01) |  | -0.89(-1.18,-0.61) |
| **Timor-Leste** | 735.71(529.22,984.83) | 93.99(70.06,124.84) |  | 1228.86(898.26,1620.44) | 93.46(69.68,122.23) |  | 0.04(0.02,0.05) |
| **Togo** | 12808.9(9219.1,16858.27) | 388.18(286.19,506.67) |  | 27919.96(20551.39,36581.68) | 353.02(263.4,461.58) |  | -0.89(-1.08,-0.69) |
| **Tokelau** | 4.33(3.17,5.76) | 299.84(221.51,401.58) |  | 3.92(2.91,5.24) | 302.98(224.18,404.13) |  | 0.04(0.01,0.07) |
| **Tonga** | 311.98(227.78,422.67) | 335.46(246.41,443.13) |  | 336.61(248,453.73) | 337.62(248.38,456.95) |  | 0(-0.04,0.03) |
| **Trinidad and Tobago** | 1577.27(1171.4,2106.45) | 127.31(96.81,168.54) |  | 1685.3(1310.75,2139.23) | 118.97(92.3,151.49) |  | -0.31(-0.39,-0.23) |
| **Tunisia** | 6898.61(4952.45,9278.57) | 82.77(60.92,110.97) |  | 9982.25(7410.57,13495.31) | 81.84(60.06,110.21) |  | -0.06(-0.07,-0.04) |
| **Turkey** | 29057.43(21300.4,38712.65) | 48.33(36.32,63.69) |  | 44732.39(33331.81,58935.83) | 48.69(36.39,64.08) |  | 0.07(-0.12,0.25) |
| **Turkmenistan** | 1910.53(1407.49,2552.37) | 53.9(40.87,71.04) |  | 2787.84(2093.22,3673.92) | 52.15(39.29,68.8) |  | -0.2(-0.24,-0.15) |
| **Tuvalu** | 24.36(17.39,33.08) | 264.89(192.08,356.54) |  | 33.86(24.38,45.75) | 276.39(200.67,374.4) |  | 0.09(0.07,0.11) |
| **Uganda** | 95444.85(70119.34,125795.44) | 621.45(470.9,807.76) |  | 322336.06(272137.71,376202.58) | 772.73(648.37,918.74) |  | 0.29(0.06,0.52) |
| **Ukraine** | 34221.49(26156.93,44845.24) | 62.84(47.94,81.46) |  | 19970.56(15198.6,26626.45) | 44.54(34.14,58.7) |  | -1.89(-2.2,-1.58) |
| **United Arab Emirates** | 2241.52(1575.68,3129.28) | 91.41(66.95,123.16) |  | 12630.14(8740.28,18215.57) | 88.31(64.98,118.36) |  | -0.03(-0.12,0.05) |
| **United Kingdom** | 42822.55(32076.96,56785.19) | 74.15(55.32,98.34) |  | 46177.55(34989.58,61516.36) | 73.32(54.45,97.81) |  | 0.07(0.02,0.12) |
| **United Republic of Tanzania** | 253579.44(207882.12,303022.52) | 1022.81(838.78,1220.09) |  | 294104.1(229936.94,364903.47) | 548.76(428.91,681.18) |  | -2.83(-3.19,-2.46) |
| **United States of America** | 189596.3(143235.73,252475.08) | 70.09(53.19,92.23) |  | 234505.92(179454.97,308635.29) | 73.96(55.71,97.89) |  | 0.2(-0.03,0.43) |
| **United States Virgin Islands** | 167.97(128.92,219.86) | 155.96(118.35,203.81) |  | 139.04(106.83,179.45) | 153.87(116.4,202.5) |  | -0.01(-0.03,0.01) |
| **Uruguay** | 3863.63(2850.21,5119.81) | 128.05(93.98,170.1) |  | 3858.44(3165.5,4642.16) | 117.91(96.71,141.43) |  | 0.03(-0.09,0.15) |
| **Uzbekistan** | 10936.45(8156.64,14338.17) | 55.54(42.65,72.54) |  | 18499.13(13896.85,24357.86) | 50.65(38.47,66.57) |  | -0.55(-0.62,-0.47) |
| **Vanuatu** | 443(320.72,587.9) | 305.71(226.03,405.42) |  | 911.5(664.19,1233.77) | 305.37(225.29,411.09) |  | 0(-0.03,0.03) |
| **Venezuela (Bolivarian Republic of)** | 33014.5(24031.66,43905.9) | 167.37(124.29,219.99) |  | 52914.28(40433.17,68102.83) | 186.66(141.1,239.7) |  | 0.42(0.35,0.5) |
| **Viet Nam** | 51713.66(36982.42,70162.47) | 75.6(55.86,100.44) |  | 84541.13(62176.82,113153.61) | 78.22(57.7,105.13) |  | 0.09(0.06,0.12) |
| **Yemen** | 19058.54(13766.45,25448.66) | 170.75(123.51,227.34) |  | 53433.95(38589.07,71686.51) | 168.91(122.38,224.84) |  | -0.46(-0.62,-0.29) |
| **Zambia** | 63740.36(49489.87,79199.06) | 852.68(664.99,1058.44) |  | 134911.02(103875.83,170399.99) | 736.58(572.75,920.96) |  | -1.12(-1.44,-0.8) |
| **Zimbabwe** | 41664.17(30633.66,55532.4) | 446.3(335.31,588.24) |  | 63116.98(49634,80801.16) | 419.57(329.82,528.34) |  | -0.2(-0.24,-0.15) |

**Note:** age-standardized incidence rate (ASIR), estimated annual percentage change (EAPC), 95% uncertainty interval (UI), 95% confidence interval (CI), social development index (SDI).

**Supplementary Table 2** The death cases and ASDR of syphilis in 1990 and 2019, and its temporal trends from 1990 to 2019

|  | **1990** | |  | **2019** | |  | **1990-2019** |
| --- | --- | --- | --- | --- | --- | --- | --- |
|  | **Death cases** | **ASDR per 100,000** |  | **Death cases** | **ASDR per 100,000** |  | **EAPC** |
|  | **No.(95% UI)** | **No.(95% UI)** |  | **No.(95% UI)** | **No.(95% UI)** |  | **No.(95% CI)** |
| Afghanistan | 125.22(35.98,291.83) | 0.53(0.16,1.21) |  | 374.66(113.59,861.97) | 0.55(0.18,1.23) |  | -0.26(0.14,-0.67) |
| Albania | 10.53(3.23,23.74) | 0.26(0.09,0.59) |  | 2.57(0.85,5.55) | 0.15(0.04,0.32) |  | -2.31(-2.07,-2.55) |
| Algeria | 165.86(50.68,382.48) | 0.46(0.14,1.04) |  | 83.7(27.16,180.44) | 0.2(0.07,0.43) |  | -3.32(-2.54,-4.09) |
| American Samoa | 2.65(0.86,5.34) | 3.27(1.09,6.56) |  | 1.19(0.41,2.34) | 2.35(0.82,4.61) |  | -1.17(-1.07,-1.27) |
| Andorra | 0.04(0.01,0.08) | 0.14(0.04,0.31) |  | 0.03(0.01,0.06) | 0.1(0.03,0.23) |  | -0.67(-0.39,-0.96) |
| Angola | 1405.76(452.13,3083.12) | 6.49(2.41,13.71) |  | 2348.45(770.36,5042.56) | 4.6(1.56,9.73) |  | -1.1(-0.93,-1.28) |
| Antigua and Barbuda | 0.07(0.06,0.09) | 0.13(0.1,0.15) |  | 0.04(0.03,0.05) | 0.04(0.03,0.05) |  | -2.65(-1.96,-3.33) |
| Argentina | 63.69(54.11,73.8) | 0.19(0.16,0.22) |  | 25.24(19.37,32.83) | 0.07(0.05,0.09) |  | -3.43(-2.89,-3.98) |
| Armenia | 5.27(1.08,6.51) | 0.18(0.04,0.22) |  | 0.05(0.02,0.48) | 0(0,0.01) |  | -20.29(-18.09,-22.44) |
| Australia | 4.02(3.18,4.8) | 0.02(0.02,0.03) |  | 2.36(1.72,3.18) | 0.01(0,0.01) |  | -3.74(-2.6,-4.87) |
| Austria | 1.38(0.6,1.71) | 0.01(0.01,0.02) |  | 0.17(0.13,0.35) | 0(0,0) |  | -4.41(-2.56,-6.22) |
| Azerbaijan | 35.61(15.13,70.71) | 0.45(0.2,0.84) |  | 18.69(5.81,41.21) | 0.26(0.08,0.59) |  | -2.52(-2.01,-3.02) |
| Bahamas | 0.08(0.06,0.09) | 0.04(0.03,0.04) |  | 0.07(0.05,0.09) | 0.02(0.01,0.02) |  | -1.67(-1.36,-1.98) |
| Bahrain | 1.48(0.43,3.46) | 0.24(0.07,0.54) |  | 1.44(0.51,3.08) | 0.23(0.08,0.48) |  | 0.04(0.44,-0.36) |
| Bangladesh | 5322.9(1691.96,11391.8) | 3.25(1.28,6.51) |  | 1954(636.89,4228.72) | 1.54(0.52,3.3) |  | -3.42(-3.14,-3.7) |
| Barbados | 0.28(0.23,0.34) | 0.12(0.1,0.15) |  | 0.11(0.09,0.15) | 0.04(0.03,0.05) |  | -3.04(-2.58,-3.5) |
| Belarus | 8.48(4.82,10.27) | 0.07(0.04,0.09) |  | 0.63(0.34,1.7) | 0.01(0,0.01) |  | -11.1(-10.25,-11.94) |
| Belgium | 2.27(0.94,3.47) | 0.02(0.01,0.02) |  | 0.11(0.08,0.41) | 0(0,0) |  | -10.9(-10.13,-11.66) |
| Belize | 0.07(0.05,0.08) | 0.03(0.02,0.03) |  | 0.03(0.03,0.04) | 0.01(0.01,0.01) |  | -2.88(-2.43,-3.33) |
| Benin | 178.6(56.8,384.81) | 1.66(0.56,3.5) |  | 209.35(69.14,444.52) | 0.89(0.3,1.87) |  | -3.77(-3.2,-4.33) |
| Bermuda | 0.02(0.02,0.02) | 0.03(0.03,0.04) |  | 0.01(0.01,0.01) | 0.01(0.01,0.01) |  | -3.41(-2.71,-4.1) |
| Bhutan | 34.99(10.67,78.53) | 3.4(1.07,7.58) |  | 20.39(5.87,47.13) | 3.15(0.91,7.29) |  | -0.89(-0.55,-1.22) |
| Bolivia (Plurinational State of) | 383.43(115.21,854.97) | 3.4(1.06,7.5) |  | 214.49(75.54,430.03) | 1.4(0.51,2.79) |  | -4.23(-3.82,-4.64) |
| Bosnia and Herzegovina | 6.5(2,14.45) | 0.2(0.06,0.44) |  | 1.55(0.53,3.39) | 0.11(0.03,0.25) |  | -1.93(-1.27,-2.59) |
| Botswana | 139.9(54.43,274.23) | 7.44(2.97,14.54) |  | 75.99(24.85,166.99) | 3.31(1.09,7.24) |  | -4.38(-3.07,-5.66) |
| Brazil | 492.02(401.5,613.53) | 0.31(0.26,0.39) |  | 250.75(207.25,300.75) | 0.15(0.13,0.19) |  | -1.79(-1.02,-2.55) |
| Brunei Darussalam | 0.59(0.22,1.21) | 0.23(0.11,0.41) |  | 0.51(0.18,1.1) | 0.17(0.06,0.36) |  | -0.81(-0.57,-1.06) |
| Bulgaria | 0.53(0.43,0.65) | 0.01(0,0.01) |  | 0.13(0.09,0.2) | 0(0,0) |  | -4.86(-3.14,-6.55) |
| Burkina Faso | 463.09(150.54,983.2) | 2.22(0.76,4.62) |  | 742.3(260.5,1550.99) | 1.69(0.6,3.51) |  | -0.32(0,-0.64) |
| Burundi | 99.67(41.08,194.21) | 1.24(0.54,2.23) |  | 90.49(38.42,177.43) | 0.61(0.31,1.11) |  | -2.9(-2.56,-3.24) |
| Cabo Verde | 7.8(2.5,17.35) | 1.39(0.45,3.08) |  | 3.08(0.96,6.98) | 0.59(0.18,1.35) |  | -4.03(-3.53,-4.54) |
| Cambodia | 44.58(14.53,100.52) | 0.25(0.1,0.51) |  | 16.96(6.42,36.09) | 0.1(0.04,0.21) |  | -5.35(-4.52,-6.18) |
| Cameroon | 1628.06(547.66,3382.33) | 7.45(2.54,15.49) |  | 1343.8(419.75,2970.78) | 3.13(0.98,6.92) |  | -2.48(-1.98,-2.99) |
| Canada | 5.55(2.18,6.9) | 0.02(0.01,0.02) |  | 1.19(0.76,1.56) | 0(0,0) |  | -6.3(-5.48,-7.11) |
| Central African Republic | 361.61(120.65,765.48) | 6.71(2.44,13.69) |  | 465.91(161.28,966.26) | 5.29(1.96,10.6) |  | -1.81(-1.54,-2.09) |
| Chad | 803.11(260.91,1713.99) | 5.47(1.83,11.65) |  | 1257.91(408.34,2615.16) | 3.4(1.11,7.07) |  | -2.25(-1.84,-2.66) |
| Chile | 9.46(7.5,10.86) | 0.08(0.06,0.1) |  | 1.46(0.96,3.82) | 0.01(0.01,0.02) |  | -5.7(-4.74,-6.65) |
| China | 4617.04(1617.87,9643.61) | 0.41(0.15,0.84) |  | 1949.73(687.24,4295.48) | 0.26(0.08,0.58) |  | -0.9(-0.58,-1.22) |
| Colombia | 79(65.8,94.25) | 0.2(0.17,0.24) |  | 23.78(16.38,33.87) | 0.06(0.04,0.08) |  | -3.98(-3.69,-4.27) |
| Comoros | 44.48(14.44,99.61) | 5.07(1.77,11.21) |  | 16.89(5.58,37.32) | 2.18(0.74,4.75) |  | -3.69(-3.39,-3.99) |
| Congo | 191(63.01,399.77) | 4.34(1.56,8.82) |  | 178.22(60.37,373.81) | 2.65(0.93,5.55) |  | -1.98(-1.72,-2.24) |
| Cook Islands | 0.51(0.16,1.11) | 2.19(0.68,4.8) |  | 0.17(0.07,0.28) | 1.29(0.56,2.15) |  | -1.72(-1.46,-1.98) |
| Costa Rica | 2.44(2.05,2.9) | 0.08(0.07,0.1) |  | 2.06(1.2,2.86) | 0.05(0.03,0.06) |  | -1.22(-0.2,-2.23) |
| Croatia | 0.24(0.15,0.29) | 0(0,0.01) |  | 0.04(0.03,0.07) | 0(0,0) |  | -5.16(-4.08,-6.23) |
| Cuba | 0.75(0.54,0.84) | 0.01(0.01,0.01) |  | 1.99(1.53,2.54) | 0.02(0.01,0.02) |  | 3.18(4.58,1.8) |
| Cyprus | 0.85(0.24,2.23) | 0.13(0.04,0.34) |  | 0.43(0.16,0.95) | 0.06(0.02,0.13) |  | -3.06(-2.73,-3.4) |
| Czechia | 2.1(0.49,2.7) | 0.02(0,0.02) |  | 0.22(0.15,0.31) | 0(0,0) |  | -7.65(-6.15,-9.13) |
| Côte d'Ivoire | 1107.22(376.31,2317.71) | 4.19(1.48,8.72) |  | 944.83(306.37,2080.74) | 2.26(0.74,4.96) |  | -2.07(-1.76,-2.36) |
| Democratic People's Republic of Korea | 110.39(31.56,280.96) | 0.33(0.1,0.83) |  | 46.74(15.22,100.46) | 0.27(0.08,0.6) |  | -0.56(-0.28,-0.83) |
| Democratic Republic of the Congo | 6790.22(2304.18,13479.37) | 8(2.88,15.54) |  | 5400.49(1907.24,11413.13) | 3.97(1.46,8.33) |  | -3.24(-2.87,-3.61) |
| Denmark | 0.48(0.29,0.63) | 0.01(0,0.01) |  | 0.37(0.15,0.51) | 0(0,0) |  | -1.89(-0.51,-3.24) |
| Djibouti | 24.11(8.51,49.11) | 2.77(1.14,5.4) |  | 33.63(12.34,70.08) | 2.18(0.86,4.36) |  | -1.11(-0.81,-1.4) |
| Dominica | 1.42(0.52,2.98) | 1.76(0.66,3.66) |  | 0.74(0.27,1.6) | 1.73(0.56,3.87) |  | 0.21(0.53,-0.11) |
| Dominican Republic | 182.6(62.62,389.33) | 1.75(0.68,3.53) |  | 197.41(72.25,410.08) | 1.81(0.67,3.75) |  | -0.52(-0.09,-0.96) |
| Ecuador | 245.98(74.57,550.68) | 1.73(0.53,3.86) |  | 169.76(49.43,387.77) | 1.01(0.29,2.3) |  | -1.98(-1.86,-2.11) |
| Egypt | 184.87(55.35,423.35) | 0.21(0.06,0.47) |  | 123.1(32.68,301.68) | 0.12(0.03,0.3) |  | -2.22(-2.03,-2.41) |
| El Salvador | 25.89(7.97,59.23) | 0.34(0.11,0.76) |  | 8.76(2.67,20.07) | 0.16(0.05,0.37) |  | -3.21(-2.9,-3.52) |
| Equatorial Guinea | 89.67(28.99,193.84) | 8.78(2.98,18.55) |  | 151.41(53.61,307.31) | 8.23(2.92,16.67) |  | -0.12(-0.01,-0.23) |
| Eritrea | 131.24(46.35,273.24) | 2.75(1.17,5.34) |  | 109.85(38.67,228.59) | 1.28(0.49,2.54) |  | -2.4(-2.03,-2.78) |
| Estonia | 3.69(0.1,4.85) | 0.18(0.01,0.24) |  | 0.02(0.01,0.03) | 0(0,0) |  | -13.29(-9.48,-16.94) |
| Eswatini | 121.33(44.06,246.68) | 8.36(3.18,16.6) |  | 38.41(12.98,81.13) | 2.78(0.97,5.81) |  | -3.38(-3.06,-3.71) |
| Ethiopia | 10897.8(3600.03,23248.29) | 10.12(3.77,20.57) |  | 4348.44(1508.91,9362.13) | 2.65(1,5.64) |  | -5.01(-4.75,-5.27) |
| Fiji | 13.15(4.37,27.46) | 1.47(0.52,3.03) |  | 11.97(3.62,26.4) | 1.42(0.43,3.13) |  | -0.17(0.02,-0.36) |
| Finland | 0.63(0.11,0.82) | 0.01(0,0.01) |  | 0.04(0.03,0.07) | 0(0,0) |  | -7.86(-6.62,-9.08) |
| France | 15.24(9.79,18.41) | 0.02(0.01,0.02) |  | 3.64(2.78,5.42) | 0(0,0) |  | -5.59(-4.97,-6.2) |
| Gabon | 77.22(26.81,171.79) | 4.37(1.63,9.36) |  | 42.39(14.04,92.51) | 2.12(0.71,4.58) |  | -3.78(-3.24,-4.32) |
| Gambia | 85.83(26.52,190.98) | 3.83(1.22,8.45) |  | 85.54(27.8,189.74) | 2.53(0.83,5.59) |  | -2.39(-1.99,-2.79) |
| Georgia | 2.06(0.7,2.59) | 0.03(0.01,0.04) |  | 0.3(0.21,0.41) | 0.01(0,0.01) |  | -1.69(0.94,-4.25) |
| Germany | 24.57(14.77,29.26) | 0.02(0.01,0.02) |  | 5.9(4.54,8.13) | 0(0,0) |  | -6.02(-5.17,-6.87) |
| Ghana | 554.28(173.61,1293.38) | 1.99(0.66,4.57) |  | 747.87(247.46,1652.11) | 1.85(0.63,4.07) |  | -0.77(-0.24,-1.29) |
| Greece | 0.83(0.41,1.03) | 0.01(0,0.01) |  | 0.1(0.08,0.23) | 0(0,0) |  | -6.26(-5.27,-7.25) |
| Greenland | 0.08(0.02,0.17) | 0.14(0.05,0.3) |  | 0.04(0.01,0.09) | 0.1(0.03,0.23) |  | -1.19(-1.05,-1.34) |
| Grenada | 0.11(0.09,0.14) | 0.15(0.12,0.18) |  | 0.04(0.03,0.05) | 0.04(0.03,0.05) |  | -3.3(-2.81,-3.78) |
| Guam | 4.49(1.49,9) | 2.5(0.83,5.01) |  | 3.72(1.18,7.68) | 2.37(0.75,4.89) |  | -0.19(-0.13,-0.25) |
| Guatemala | 7.89(6.39,9.4) | 0.12(0.08,0.14) |  | 2.76(2.08,3.68) | 0.02(0.02,0.03) |  | -5.76(-5.38,-6.14) |
| Guinea | 605.55(194.61,1289.81) | 4.19(1.4,8.86) |  | 545.62(166.24,1170.95) | 2.42(0.75,5.18) |  | -2.53(-1.97,-3.08) |
| Guinea-Bissau | 224.31(68.46,485.42) | 10.38(3.26,22.3) |  | 125.26(39.7,271.55) | 4.24(1.36,9.18) |  | -3.81(-2.65,-4.96) |
| Guyana | 2.91(2.15,3.94) | 0.36(0.28,0.47) |  | 0.62(0.43,0.87) | 0.09(0.06,0.12) |  | -3.37(-2.84,-3.9) |
| Haiti | 595.73(206.57,1283.74) | 5.37(2.03,11.12) |  | 743.22(276.75,1506) | 4.79(1.81,9.67) |  | -0.6(-0.17,-1.02) |
| Honduras | 34.07(10.61,75.67) | 0.42(0.15,0.91) |  | 23.78(7.73,53.38) | 0.23(0.08,0.49) |  | -3.37(-2.92,-3.83) |
| Hungary | 1.85(0.47,2.31) | 0.01(0,0.02) |  | 0.11(0.09,0.17) | 0(0,0) |  | -6.39(-4.31,-8.42) |
| Iceland | 0.06(0.03,0.08) | 0.02(0.01,0.03) |  | 0.01(0.01,0.03) | 0(0,0.01) |  | -8.34(-7.8,-8.89) |
| India | 15968.48(5158.35,35216.57) | 1.31(0.44,2.85) |  | 7175.64(2417.34,15408.55) | 0.63(0.21,1.36) |  | -3.49(-2.58,-4.38) |
| Indonesia | 3686.85(1137.14,8379.54) | 1.66(0.54,3.73) |  | 3642.15(1125.35,7981.24) | 1.96(0.6,4.3) |  | 0.94(1.29,0.59) |
| Iran (Islamic Republic of) | 122.68(38.21,293.54) | 0.15(0.05,0.35) |  | 76.51(32.34,151.95) | 0.12(0.05,0.23) |  | -0.98(-0.79,-1.17) |
| Iraq | 103.23(31.26,231.63) | 0.32(0.11,0.7) |  | 111.71(34.55,245.3) | 0.24(0.08,0.53) |  | -0.85(-0.68,-1.03) |
| Ireland | 0.4(0.19,0.51) | 0.01(0,0.01) |  | 0.03(0.03,0.05) | 0(0,0) |  | -8.35(-7.62,-9.06) |
| Israel | 0.46(0.27,0.56) | 0.01(0.01,0.01) |  | 0.11(0.09,0.2) | 0(0,0) |  | -8.67(-7.21,-10.1) |
| Italy | 20.27(18.56,31.95) | 0.03(0.03,0.04) |  | 4.52(3.58,11.5) | 0(0,0.01) |  | -7.39(-6.9,-7.87) |
| Jamaica | 1.11(0.91,1.35) | 0.05(0.04,0.06) |  | 0.96(0.61,1.35) | 0.03(0.02,0.05) |  | 0.02(1.07,-1.02) |
| Japan | 26.6(19.57,28.62) | 0.02(0.01,0.02) |  | 10.16(8.58,14.03) | 0(0,0) |  | -4.67(-4.13,-5.2) |
| Jordan | 27.47(8.21,64.31) | 0.42(0.13,0.99) |  | 39.55(11.93,91.29) | 0.33(0.1,0.77) |  | -0.96(-0.74,-1.17) |
| Kazakhstan | 7.67(6.31,9) | 0.05(0.04,0.06) |  | 1.85(1.33,2.87) | 0.01(0.01,0.02) |  | -6.9(-5.29,-8.49) |
| Kenya | 1575.18(514.37,3383.69) | 3.49(1.27,7.25) |  | 944.58(324.57,2014.66) | 1.54(0.57,3.17) |  | -2.85(-2.53,-3.16) |
| Kiribati | 4.21(1.34,8.94) | 3.47(1.13,7.34) |  | 5.01(1.57,10.48) | 3.47(1.09,7.25) |  | 0.31(0.47,0.15) |
| Kuwait | 0.04(0.03,0.05) | 0(0,0.01) |  | 0.05(0.04,0.06) | 0(0,0) |  | -4.85(-3.58,-6.11) |
| Kyrgyzstan | 9.62(6.68,14.93) | 0.17(0.12,0.26) |  | 0.41(0.3,0.69) | 0.01(0,0.01) |  | -12.78(-10.49,-15.01) |
| Lao People's Democratic Republic | 43.51(13.24,96.29) | 0.6(0.21,1.27) |  | 33.68(10.26,77.42) | 0.42(0.14,0.97) |  | -1.23(-1.02,-1.45) |
| Latvia | 3.25(0.64,4.07) | 0.1(0.02,0.12) |  | 0.03(0.02,0.15) | 0(0,0.01) |  | -19.62(-17.21,-21.96) |
| Lebanon | 8.42(2.56,19.02) | 0.16(0.05,0.37) |  | 7.66(2.28,17.75) | 0.15(0.04,0.35) |  | -0.99(-0.7,-1.27) |
| Lesotho | 150.68(52.2,304.11) | 5.52(1.99,10.86) |  | 68.5(22.74,151.68) | 3.17(1.08,6.99) |  | -1.34(-1.1,-1.57) |
| Liberia | 350.5(111.88,757.67) | 9.28(3.01,19.95) |  | 518.44(186.3,1023.87) | 8.09(2.91,15.97) |  | 0.32(0.9,-0.26) |
| Libya | 13.9(4.25,31.52) | 0.2(0.06,0.44) |  | 7.47(2.57,16.11) | 0.19(0.06,0.4) |  | 0.46(0.81,0.11) |
| Lithuania | 1.98(0.37,2.45) | 0.05(0.01,0.06) |  | 0.03(0.02,0.08) | 0(0,0) |  | -12.91(-10.81,-14.97) |
| Luxembourg | 0.4(0.28,0.51) | 0.08(0.06,0.1) |  | 0.1(0.07,0.17) | 0.01(0.01,0.02) |  | -6.77(-5.94,-7.6) |
| Madagascar | 1932.11(682.06,3905.61) | 7.98(2.92,15.94) |  | 2566.56(953.54,4979.69) | 6.39(2.44,12.31) |  | -1.17(-0.71,-1.63) |
| Malawi | 1101.29(397.79,2225.01) | 5.3(2.08,10.48) |  | 1329.29(485.97,2709.38) | 5.19(1.96,10.46) |  | -0.8(0.01,-1.61) |
| Malaysia | 33.7(11.4,73.17) | 0.15(0.06,0.31) |  | 19.49(7.68,41.43) | 0.07(0.03,0.16) |  | -2.93(-2.63,-3.23) |
| Maldives | 1.29(0.41,2.86) | 0.36(0.15,0.76) |  | 0.66(0.24,1.42) | 0.17(0.06,0.35) |  | -2.56(-2.28,-2.85) |
| Mali | 1158.81(348.79,2606.05) | 5.7(1.79,12.72) |  | 2084.31(720.99,4201.65) | 4.62(1.6,9.29) |  | -0.07(0.28,-0.43) |
| Malta | 0.03(0.03,0.04) | 0.01(0.01,0.01) |  | 0.01(0.01,0.01) | 0(0,0) |  | -5.36(-4.72,-5.99) |
| Marshall Islands | 1.99(0.63,4.08) | 2.76(0.88,5.62) |  | 1.58(0.53,3.2) | 2.67(0.9,5.38) |  | 0.13(0.4,-0.13) |
| Mauritania | 216.13(71.08,458.39) | 5.18(1.75,10.93) |  | 153.35(49.66,329.12) | 2.93(0.96,6.29) |  | -1.57(-1.34,-1.81) |
| Mauritius | 0.14(0.08,0.17) | 0.02(0.01,0.02) |  | 0.07(0.05,0.08) | 0(0,0.01) |  | -1.63(0.34,-3.55) |
| Mexico | 73.67(65.74,81.8) | 0.09(0.08,0.1) |  | 24.29(20.68,28.58) | 0.02(0.02,0.02) |  | -3.9(-2.88,-4.9) |
| Micronesia (Federated States of) | 6.38(2.1,13.8) | 3.86(1.3,8.28) |  | 2.62(0.96,5.05) | 2.79(1.02,5.38) |  | -0.91(-0.72,-1.1) |
| Monaco | 0.02(0.01,0.05) | 0.13(0.04,0.3) |  | 0.01(0.01,0.03) | 0.08(0.03,0.18) |  | -1.85(-1.7,-2) |
| Mongolia | 47.84(12.95,106.95) | 1.37(0.37,3.07) |  | 80.25(21.12,171.24) | 2.07(0.55,4.42) |  | 2.38(3,1.77) |
| Montenegro | 0.86(0.3,1.89) | 0.17(0.06,0.39) |  | 0.52(0.21,1.04) | 0.14(0.05,0.3) |  | -0.91(-0.79,-1.04) |
| Morocco | 431.31(133.34,977.33) | 1.15(0.36,2.6) |  | 213.58(61.43,494.31) | 0.74(0.21,1.7) |  | -3.92(-2.82,-5) |
| Mozambique | 4327.59(1529.49,8811.91) | 16.18(5.96,32.47) |  | 2490.39(879.21,5160.19) | 4.87(1.81,9.86) |  | -3.96(-3.66,-4.26) |
| Myanmar | 516.57(154.54,1205.77) | 0.85(0.26,1.95) |  | 509.1(169.09,1110.5) | 1.02(0.34,2.22) |  | 0.67(0.88,0.46) |
| Namibia | 84.38(27.54,177.29) | 3.85(1.38,7.92) |  | 71.2(23.89,149.19) | 2.41(0.83,5) |  | -3.05(-2.15,-3.94) |
| Nauru | 0.41(0.13,0.92) | 2.06(0.65,4.66) |  | 0.37(0.11,0.81) | 2.57(0.79,5.71) |  | 0.96(1.25,0.67) |
| Nepal | 689.8(209.15,1547.03) | 1.87(0.62,4.1) |  | 415.2(127.6,888.59) | 1.41(0.44,3.02) |  | -1.1(-1.03,-1.16) |
| Netherlands | 4.53(1.76,5.47) | 0.02(0.01,0.03) |  | 1.1(0.81,1.51) | 0(0,0) |  | -7.3(-6.06,-8.52) |
| New Zealand | 0.34(0.15,0.42) | 0.01(0,0.01) |  | 0.1(0.08,0.13) | 0(0,0) |  | -3.89(-1.95,-5.78) |
| Nicaragua | 30.54(9.32,66.37) | 0.46(0.15,0.98) |  | 9.67(3.19,20.88) | 0.15(0.05,0.33) |  | -5.08(-4,-6.14) |
| Niger | 167.48(55.99,368.48) | 0.89(0.35,1.86) |  | 189.41(63.51,406.49) | 0.36(0.13,0.76) |  | -4.13(-3.59,-4.67) |
| Nigeria | 4283.12(1317.42,9409.61) | 2.24(0.74,4.89) |  | 8785.56(2805.97,20068.23) | 2.46(0.79,5.61) |  | 0.9(1.25,0.54) |
| Niue | 0.06(0.02,0.13) | 2.29(0.72,4.98) |  | 0.03(0.01,0.07) | 2.56(0.83,5.62) |  | 0.57(0.67,0.47) |
| North Macedonia | 2.6(0.78,5.9) | 0.15(0.05,0.35) |  | 1.51(0.49,3.36) | 0.14(0.04,0.31) |  | -0.22(-0.02,-0.43) |
| Northern Mariana Islands | 1.22(0.41,2.57) | 2.05(0.7,4.29) |  | 0.48(0.17,0.95) | 2.03(0.69,4.01) |  | 0.42(0.66,0.18) |
| Norway | 0.44(0.34,1.58) | 0.01(0.01,0.02) |  | 3.62(0.56,4.52) | 0.03(0.01,0.04) |  | 7.26(8.43,6.1) |
| Oman | 3.14(0.97,7.29) | 0.1(0.04,0.23) |  | 2.12(0.77,4.61) | 0.06(0.02,0.12) |  | -3.07(-2.71,-3.43) |
| Pakistan | 4783.55(1394.67,10855.22) | 2.14(0.66,4.82) |  | 4707.09(1491.13,10366.92) | 1.5(0.49,3.29) |  | -0.33(0.07,-0.73) |
| Palau | 0.43(0.14,0.93) | 2.42(0.77,5.26) |  | 0.27(0.1,0.53) | 2.97(1.06,5.82) |  | 1.08(1.24,0.93) |
| Palestine | 9.65(2.96,21.59) | 0.22(0.07,0.49) |  | 10.96(3.42,23.51) | 0.19(0.06,0.39) |  | -0.49(-0.37,-0.61) |
| Panama | 0.3(0.25,0.37) | 0.01(0.01,0.02) |  | 0.88(0.62,1.24) | 0.02(0.02,0.03) |  | 5.07(6.55,3.6) |
| Papua New Guinea | 474.43(159.52,1016.62) | 6.76(2.32,14.46) |  | 877.42(280.28,1832.15) | 5.67(1.87,11.8) |  | -1.29(-1.01,-1.56) |
| Paraguay | 200.4(64.37,419.71) | 3.28(1.07,6.86) |  | 221.03(78.6,442.73) | 3.62(1.29,7.25) |  | 0.48(0.75,0.22) |
| Peru | 547.73(166.55,1173.35) | 1.76(0.56,3.73) |  | 192.43(61.44,436.9) | 0.62(0.2,1.41) |  | -5.12(-4.7,-5.54) |
| Philippines | 781.4(238.98,1713.45) | 0.8(0.25,1.74) |  | 1130.28(340.24,2514) | 0.9(0.28,1.99) |  | 0.33(0.53,0.12) |
| Poland | 8.63(3.83,9.51) | 0.02(0.01,0.02) |  | 1.11(0.78,1.92) | 0(0,0) |  | -7.46(-6.52,-8.38) |
| Portugal | 4.28(3.56,5.07) | 0.04(0.04,0.05) |  | 0.9(0.67,1.29) | 0.01(0,0.01) |  | -7.26(-4.99,-9.48) |
| Puerto Rico | 0.73(0.64,0.96) | 0.02(0.02,0.03) |  | 0.85(0.45,1.2) | 0.01(0.01,0.02) |  | -1.64(-0.52,-2.75) |
| Qatar | 1.33(0.38,3.02) | 0.27(0.08,0.6) |  | 2.59(0.82,5.62) | 0.19(0.06,0.42) |  | -1.54(-1.35,-1.72) |
| Republic of Korea | 62.43(20.17,139.95) | 0.19(0.06,0.43) |  | 42.9(15.03,91.3) | 0.21(0.07,0.45) |  | 1(1.27,0.74) |
| Republic of Moldova | 1.57(0.96,1.86) | 0.04(0.02,0.04) |  | 0.08(0.06,0.24) | 0(0,0.01) |  | -9.03(-8.1,-9.95) |
| Romania | 2.33(2.01,3.19) | 0.01(0.01,0.01) |  | 0.81(0.6,1.1) | 0(0,0.01) |  | -5.82(-3.4,-8.17) |
| Russian Federation | 62.47(55.18,71.15) | 0.04(0.04,0.05) |  | 21.37(16.3,27.56) | 0.02(0.01,0.02) |  | -5.47(-4.23,-6.7) |
| Rwanda | 397.43(137.41,846.81) | 3.05(1.24,5.95) |  | 285.6(108.24,603.11) | 1.78(0.71,3.65) |  | -1.83(-1.5,-2.17) |
| Saint Kitts and Nevis | 0.11(0.09,0.13) | 0.27(0.23,0.32) |  | 0.04(0.03,0.05) | 0.07(0.05,0.09) |  | -3.35(-2.58,-4.12) |
| Saint Lucia | 0.4(0.32,0.49) | 0.28(0.23,0.33) |  | 0.12(0.09,0.16) | 0.08(0.06,0.11) |  | -3.29(-2.73,-3.85) |
| Saint Vincent and the Grenadines | 0.81(0.53,1.05) | 0.81(0.5,1.02) |  | 0.15(0.11,0.19) | 0.13(0.1,0.17) |  | -3.79(-2.66,-4.92) |
| Samoa | 4.85(1.53,10.35) | 2.92(0.93,6.22) |  | 5.02(1.65,10.76) | 2.72(0.9,5.82) |  | -0.18(0.16,-0.53) |
| San Marino | 0.01(0,0.03) | 0.09(0.03,0.23) |  | 0.01(0,0.03) | 0.08(0.02,0.17) |  | -0.62(-0.38,-0.86) |
| Sao Tome and Principe | 4.79(1.5,10.48) | 2.2(0.71,4.77) |  | 3.14(0.98,6.96) | 1.4(0.44,3.09) |  | -2(-1.37,-2.63) |
| Saudi Arabia | 36.19(11.09,84.58) | 0.14(0.05,0.33) |  | 14.65(5.22,30.39) | 0.06(0.02,0.13) |  | -2.78(-2.6,-2.96) |
| Senegal | 237.26(79.41,501.94) | 1.53(0.55,3.17) |  | 169.06(55.83,358.65) | 0.77(0.26,1.63) |  | -3.88(-3.3,-4.45) |
| Serbia | 11.98(3.63,26.75) | 0.18(0.05,0.41) |  | 4.46(1.52,9.65) | 0.11(0.03,0.24) |  | -1.88(-1.69,-2.07) |
| Seychelles | 0.18(0.08,0.35) | 0.24(0.11,0.45) |  | 0.15(0.07,0.29) | 0.19(0.09,0.39) |  | -0.51(-0.38,-0.63) |
| Sierra Leone | 240.93(72.29,524.3) | 3.31(1.02,7.15) |  | 258.66(77.48,585.09) | 1.97(0.6,4.45) |  | -2.02(-1.77,-2.27) |
| Singapore | 2.57(0.31,3.26) | 0.13(0.01,0.16) |  | 0.02(0.02,0.11) | 0(0,0) |  | -17.79(-15.19,-20.31) |
| Slovakia | 4.92(1.55,10.93) | 0.13(0.04,0.28) |  | 2.88(0.94,6.39) | 0.1(0.03,0.23) |  | -0.53(-0.38,-0.68) |
| Slovenia | 0.08(0.04,0.1) | 0(0,0) |  | 0.01(0.01,0.02) | 0(0,0) |  | -5.51(-4.9,-6.11) |
| Solomon Islands | 63.99(22.65,125.95) | 9.49(3.4,18.55) |  | 103.65(43.28,179.85) | 10.42(4.37,18.04) |  | 0.96(1.31,0.62) |
| Somalia | 562.45(203.8,1167.29) | 4.52(1.84,9.31) |  | 1275.94(454.29,2610.59) | 3.5(1.39,6.83) |  | -0.21(0.17,-0.59) |
| South Africa | 3589.14(1285.31,7218.71) | 7.84(2.87,15.59) |  | 2279.39(780.7,4606.97) | 4.6(1.58,9.3) |  | -1.81(-1.32,-2.3) |
| South Sudan | 859.77(292.83,1868.05) | 7.8(2.85,16.2) |  | 1079.63(334.8,2393.05) | 6.52(2.11,14.34) |  | 0.58(0.89,0.26) |
| Spain | 30.77(24.62,36.77) | 0.06(0.05,0.08) |  | 4.29(3.21,7.43) | 0.01(0,0.01) |  | -8.16(-7.35,-8.97) |
| Sri Lanka | 23.3(8.78,48.41) | 0.14(0.06,0.29) |  | 12.45(5.49,25.67) | 0.08(0.03,0.17) |  | -2.32(-2.01,-2.64) |
| Sudan | 822.14(226.33,1979.21) | 1.8(0.5,4.32) |  | 999.94(281.24,2408.01) | 1.79(0.5,4.29) |  | -1.03(-0.52,-1.53) |
| Suriname | 9.7(2.98,21.46) | 2.24(0.7,4.94) |  | 6.26(1.88,14) | 1.44(0.42,3.23) |  | -1.74(-1.61,-1.87) |
| Sweden | 1.02(0.17,1.25) | 0.01(0,0.01) |  | 0.08(0.07,0.11) | 0(0,0) |  | -7.18(-6.34,-8.01) |
| Switzerland | 0.9(0.5,1.38) | 0.01(0.01,0.01) |  | 0.72(0.2,0.96) | 0(0,0.01) |  | -2.76(-1.24,-4.25) |
| Syrian Arab Republic | 74.13(21.3,186.21) | 0.32(0.1,0.78) |  | 20.96(7.14,44.58) | 0.19(0.06,0.4) |  | -1.05(-0.75,-1.34) |
| Taiwan (Province of China) | 2.42(2.08,2.81) | 0.02(0.01,0.02) |  | 2.75(2.06,3.57) | 0.01(0.01,0.01) |  | -1.86(-1.15,-2.58) |
| Tajikistan | 24.93(11.23,47.72) | 0.39(0.24,0.66) |  | 21.48(10.91,39.41) | 0.23(0.13,0.39) |  | -2.84(-2.36,-3.31) |
| Thailand | 153.97(52.59,336.85) | 0.3(0.1,0.66) |  | 27.75(12.87,53.78) | 0.08(0.03,0.18) |  | -6.9(-5.62,-8.17) |
| Timor-Leste | 15.11(4.63,34.08) | 0.93(0.31,2.06) |  | 12.33(4.05,27.45) | 0.69(0.24,1.52) |  | -1.12(-0.98,-1.26) |
| Togo | 164.77(51.46,373.81) | 2.19(0.74,4.9) |  | 106.73(34.3,236.93) | 0.95(0.31,2.11) |  | -5.32(-4.46,-6.17) |
| Tokelau | 0.06(0.02,0.13) | 2.18(0.71,4.52) |  | 0.03(0.01,0.06) | 1.57(0.54,3.11) |  | -0.84(-0.62,-1.05) |
| Tonga | 2.82(0.89,6.17) | 2.26(0.72,4.93) |  | 2.03(0.64,4.27) | 1.81(0.57,3.78) |  | -0.89(-0.73,-1.06) |
| Trinidad and Tobago | 0.08(0.07,0.09) | 0.01(0.01,0.01) |  | 0.17(0.12,0.24) | 0.01(0.01,0.01) |  | 0.94(2.36,-0.45) |
| Tunisia | 34.62(9.96,78.59) | 0.31(0.09,0.7) |  | 12.61(4.33,27.04) | 0.15(0.05,0.33) |  | -2.24(-2.04,-2.43) |
| Turkey | 188.44(55.89,431.89) | 0.24(0.07,0.55) |  | 61.86(21.3,138.57) | 0.13(0.04,0.29) |  | -2.23(-2.12,-2.34) |
| Turkmenistan | 1.2(0.46,1.44) | 0.05(0.02,0.06) |  | 0.14(0.09,0.27) | 0(0,0.01) |  | -11.85(-10,-13.66) |
| Tuvalu | 0.5(0.15,1.17) | 3.62(1.11,8.52) |  | 0.26(0.09,0.51) | 2.47(0.87,4.89) |  | -1.36(-1.21,-1.51) |
| Uganda | 1872.26(607.48,4075.58) | 4.55(1.64,9.64) |  | 6005.42(2301.53,11680.95) | 8.1(3.13,15.68) |  | 1.19(1.78,0.61) |
| Ukraine | 91.37(23.76,107.73) | 0.13(0.04,0.16) |  | 4.93(2.74,22.19) | 0.01(0,0.04) |  | -16.65(-13.49,-19.7) |
| United Arab Emirates | 2.73(0.86,6.32) | 0.13(0.04,0.29) |  | 2.22(1.24,4.01) | 0.06(0.02,0.12) |  | -3.91(-3.34,-4.48) |
| United Kingdom | 61.16(25.98,125.97) | 0.14(0.05,0.31) |  | 48.91(15.43,103.5) | 0.12(0.04,0.27) |  | 0.89(1.35,0.44) |
| United Republic of Tanzania | 5267.24(1940.61,10400.31) | 9.47(3.56,18.48) |  | 3837.56(1389.92,7698.28) | 4(1.48,7.91) |  | -3.35(-2.97,-3.72) |
| United States of America | 60.89(53.1,64.59) | 0.02(0.02,0.02) |  | 37.91(33.51,44.46) | 0.01(0.01,0.01) |  | -3.38(-2.86,-3.89) |
| United States Virgin Islands | 1.4(0.51,2.92) | 1.31(0.49,2.73) |  | 0.63(0.27,1.26) | 0.89(0.32,1.91) |  | -1.3(-1.24,-1.37) |
| Uruguay | 12.36(7.99,15.26) | 0.4(0.27,0.51) |  | 0.54(0.43,0.79) | 0.01(0.01,0.02) |  | -12(-10.24,-13.73) |
| Uzbekistan | 12.77(3.76,15.26) | 0.09(0.03,0.11) |  | 0.76(0.46,3.19) | 0(0,0.01) |  | -16.31(-14.25,-18.33) |
| Vanuatu | 9.94(3.33,20.55) | 3.68(1.24,7.56) |  | 17.33(6.17,33.44) | 4.7(1.7,9.04) |  | 0.4(0.53,0.28) |
| Venezuela (Bolivarian Republic of) | 16.83(13.56,20.82) | 0.08(0.06,0.09) |  | 14.25(9.48,20.6) | 0.06(0.04,0.08) |  | -2.13(-1.31,-2.95) |
| Viet Nam | 443.58(131.98,1029.61) | 0.51(0.16,1.19) |  | 227.44(71.27,489.66) | 0.35(0.11,0.75) |  | -1.88(-1.73,-2.03) |
| Yemen | 291.82(88.9,715.75) | 0.93(0.29,2.25) |  | 399.69(123.61,905.9) | 0.9(0.29,2.03) |  | 0.05(0.22,-0.12) |
| Zambia | 1124.4(398.92,2225.97) | 6.46(2.4,12.6) |  | 1348.8(486.99,2742.13) | 4.68(1.74,9.45) |  | -1.55(-1.29,-1.81) |
| Zimbabwe | 411.26(133.05,866.02) | 2.42(0.87,4.95) |  | 427.6(149.66,880.96) | 2.1(0.77,4.21) |  | -0.33(-0.16,-0.49) |

**Note:** age-standardized death rate (ASDR), estimated annual percentage change (EAPC), 95% uncertainty interval (UI), 95% confidence interval (CI), social development index (SDI).

**Supplementary Table 3** The DALYs and Age-standardized DALY rate of syphilis in 1990 and 2019, and its temporal trends from 1990 to 2019

|  | **1990** | |  | **2019** | |  | **1990-2019** |
| --- | --- | --- | --- | --- | --- | --- | --- |
|  | **DALYs** | **Age-standardized DALY rate per 100,000** |  | **DALYs** | **Age-standardized DALY rate per 100,000** |  | **EAPC** |
|  | **No.(95% UI)** | **No.(95% UI)** |  | **No.(95% UI)** | **No.(95% UI)** |  | **No.(95% CI)** |
| Afghanistan | 11031.32(3132.92,25848.09) | 45.75(13.33,106.55) |  | 33114.47(9939.9,76360.95) | 47.33(14.49,108.48) |  | -0.28(-0.69,0.14) |
| Albania | 911.8(260.4,2074.28) | 22.35(6.61,50.61) |  | 220.36(67.99,485.31) | 12.8(3.77,28.5) |  | -2.15(-2.38,-1.92) |
| Algeria | 14663.15(4447.31,33905.76) | 39.98(12.24,92.2) |  | 7325.33(2329.85,15930.61) | 17.39(5.53,37.8) |  | -3.36(-4.14,-2.57) |
| American Samoa | 234.5(75.87,472.82) | 288.15(93.94,580.01) |  | 105.65(36.61,207.46) | 208.22(72.23,408.99) |  | -1.15(-1.25,-1.05) |
| Andorra | 3.19(1.06,7.11) | 12.07(3.64,27.63) |  | 2.36(0.94,5) | 9.06(2.85,20.44) |  | -0.68(-0.96,-0.4) |
| Angola | 123000.67(38892.46,270415.59) | 534.8(178.62,1165.13) |  | 207227.02(67456.6,445885.52) | 397.21(131.75,850.47) |  | -0.93(-1.11,-0.74) |
| Antigua and Barbuda | 3.98(3.11,5.14) | 6.59(5.18,8.41) |  | 1.74(1.32,2.26) | 2.13(1.57,2.88) |  | -2.74(-3.33,-2.16) |
| Argentina | 4955.1(4169.21,5818.34) | 14.93(12.57,17.52) |  | 1802.23(1337.1,2382.66) | 4.99(3.63,6.75) |  | -3.48(-4,-2.96) |
| Armenia | 179.92(40.74,221.01) | 5.77(1.3,7.04) |  | 3.67(1.94,17.09) | 0.12(0.07,0.49) |  | -17.24(-19.18,-15.25) |
| Australia | 163.47(135.51,197.8) | 1.08(0.89,1.31) |  | 73.71(58.2,98.53) | 0.26(0.21,0.35) |  | -3.73(-4.56,-2.9) |
| Austria | 91.71(69.61,112.63) | 0.97(0.75,1.19) |  | 59(41.82,78.8) | 0.48(0.35,0.64) |  | -1.38(-1.83,-0.93) |
| Azerbaijan | 2780.84(980.4,5892.07) | 32.08(12.22,66.26) |  | 1594.3(489.12,3574.47) | 22.68(6.84,51.29) |  | -1.65(-1.97,-1.33) |
| Bahamas | 5.59(4.47,6.95) | 2.26(1.83,2.79) |  | 4.58(3.56,6) | 1.25(0.97,1.64) |  | -1.54(-1.88,-1.2) |
| Bahrain | 130.65(37.42,307.2) | 20.06(5.83,46.97) |  | 124.59(41.06,270.39) | 19.24(6.05,42.34) |  | -0.06(-0.5,0.38) |
| Bangladesh | 459870.52(137102.38,999741.99) | 254.16(81.12,544.1) |  | 168009.65(52898.83,370351.14) | 129.84(41.1,286.44) |  | -3.14(-3.43,-2.86) |
| Barbados | 18.04(14.38,22.38) | 8.23(6.45,10.37) |  | 5.93(4.56,7.71) | 2.6(1.89,3.54) |  | -3.22(-3.75,-2.69) |
| Belarus | 346.33(211.55,427.16) | 3.39(2.19,4.37) |  | 34.39(22.75,66.6) | 0.38(0.25,0.64) |  | -9.58(-10.33,-8.82) |
| Belgium | 100.86(70.42,128.83) | 0.83(0.59,1.04) |  | 45.64(32.3,60.86) | 0.31(0.22,0.42) |  | -3.04(-3.5,-2.59) |
| Belize | 5.87(4.45,7.56) | 2.31(1.8,2.91) |  | 3.11(2.5,3.97) | 0.81(0.65,1.01) |  | -3.14(-3.61,-2.67) |
| Benin | 15749.3(4942.44,34072.56) | 143.33(46.9,307) |  | 18519.51(6055.95,39375.5) | 78.22(26.19,165.48) |  | -3.73(-4.3,-3.16) |
| Bermuda | 0.98(0.82,1.18) | 1.7(1.43,2.03) |  | 0.44(0.35,0.58) | 0.59(0.46,0.79) |  | -2.74(-3.35,-2.14) |
| Bhutan | 3095.95(938.44,6959.26) | 297.46(91.32,667.86) |  | 1803.21(516.85,4163.86) | 278.64(79.81,643.45) |  | -0.86(-1.19,-0.52) |
| Bolivia (Plurinational State of) | 33818.02(10088.89,75695.18) | 295.57(89.15,659.11) |  | 18858.74(6578.05,37997.7) | 122.36(42.87,245.94) |  | -4.24(-4.66,-3.82) |
| Bosnia and Herzegovina | 568.67(171.33,1273.38) | 17.15(5.08,38.63) |  | 132.12(41.85,293.17) | 9.69(2.76,22.01) |  | -1.93(-2.58,-1.27) |
| Botswana | 12276.68(4720.6,24142.44) | 637.83(249.13,1249.15) |  | 6652.63(2150.94,14712.86) | 287.76(93.21,635.78) |  | -4.38(-5.72,-3.03) |
| Brazil | 42039.06(34085.32,52843.77) | 25.94(21.08,32.51) |  | 20250.77(16378.66,24679.21) | 12.85(10.27,15.81) |  | -1.8(-2.59,-0.99) |
| Brunei Darussalam | 49.32(16.73,104.29) | 16.03(6.38,32.01) |  | 44.56(15.47,96.87) | 14.13(4.63,31.14) |  | -0.11(-0.31,0.1) |
| Bulgaria | 29(24.45,33.92) | 0.37(0.31,0.44) |  | 12(9.11,15.73) | 0.18(0.14,0.23) |  | -3.4(-4.89,-1.88) |
| Burkina Faso | 40862.32(13187.02,87027.32) | 192.33(63.22,404.64) |  | 65745.05(22966.85,137397.46) | 148.89(52.71,310.51) |  | -0.24(-0.56,0.09) |
| Burundi | 8043.12(2971.05,16357.53) | 81.19(34.78,152.83) |  | 7164.68(2813.54,14562.63) | 39.49(17.78,74.37) |  | -2.96(-3.36,-2.55) |
| Cabo Verde | 692.69(222.22,1539.09) | 123.26(39.73,273.35) |  | 274.86(87.25,620.62) | 52.92(16.71,119.62) |  | -4.02(-4.52,-3.51) |
| Cambodia | 3836.77(1180.29,8735.69) | 19.5(6.61,43.38) |  | 1389.6(469.98,3103.69) | 7.99(2.75,17.74) |  | -5.62(-6.56,-4.68) |
| Cameroon | 144284.01(48438.44,299603.58) | 657.94(222.49,1368.31) |  | 119152.52(37222.34,263231.44) | 277.61(87.08,612.77) |  | -2.47(-2.97,-1.96) |
| Canada | 314.42(234.78,394.29) | 1.01(0.77,1.26) |  | 353.04(252.54,480.08) | 0.63(0.46,0.85) |  | -1.24(-1.51,-0.96) |
| Central African Republic | 31520.39(10055.09,67024.14) | 550.75(190.89,1158.59) |  | 40859.78(14004.01,85611.92) | 447.53(155.93,924.48) |  | -1.75(-2.04,-1.46) |
| Chad | 71098.96(22954.76,151804.7) | 481.01(158.57,1027.48) |  | 111488.07(36170.15,231677.25) | 300.76(98.38,625.26) |  | -2.23(-2.64,-1.82) |
| Chile | 480.31(407.95,553.38) | 3.76(3.19,4.32) |  | 84.1(64.81,149.31) | 0.52(0.4,0.81) |  | -5.14(-5.88,-4.4) |
| China | 395598.51(130202.04,840568.85) | 34.37(11.38,72.92) |  | 170634.75(58117.25,379775.26) | 22.51(7.03,51.27) |  | -0.77(-1.09,-0.45) |
| Colombia | 6706.92(5550.1,8014.42) | 16.02(13.42,19) |  | 1951.68(1369.89,2730.13) | 4.65(3.2,6.62) |  | -3.88(-4.15,-3.6) |
| Comoros | 3898.25(1250.19,8749.81) | 429.3(140.35,959.57) |  | 1463.14(467.05,3286.88) | 186.4(60.72,416.88) |  | -3.68(-3.99,-3.38) |
| Congo | 16692.27(5225.55,35221.2) | 361.34(122.05,751.5) |  | 15686.31(5242.38,33092.76) | 229.3(77.62,481.02) |  | -1.85(-2.12,-1.57) |
| Cook Islands | 44.86(13.79,98.53) | 194.08(59.76,426) |  | 15.18(6.54,25.14) | 114.9(49.41,190.18) |  | -1.71(-1.97,-1.45) |
| Costa Rica | 186.87(154.53,226.91) | 5.58(4.72,6.6) |  | 122.39(89.99,160.31) | 2.91(2.1,3.92) |  | -1.6(-2.51,-0.67) |
| Croatia | 12.76(10.17,15.37) | 0.26(0.21,0.31) |  | 5.54(4.15,7.73) | 0.12(0.09,0.17) |  | -2.36(-2.81,-1.9) |
| Cuba | 58.86(47.18,78.13) | 0.56(0.45,0.72) |  | 116.86(91.34,148.61) | 0.94(0.73,1.21) |  | 2.23(1.16,3.31) |
| Cyprus | 74.9(20.27,197.19) | 11.23(3,29.67) |  | 37.19(12.79,83.08) | 4.85(1.53,11.09) |  | -3.08(-3.41,-2.74) |
| Czechia | 70.72(27.8,86.43) | 0.59(0.28,0.71) |  | 16.52(12.59,22.42) | 0.13(0.1,0.18) |  | -4.05(-4.99,-3.1) |
| Côte d'Ivoire | 97994.72(33090.45,205104.6) | 368.19(127.03,768.79) |  | 83723.02(27048.35,184368.19) | 199.81(65,439.43) |  | -2.04(-2.33,-1.74) |
| Democratic People's Republic of Korea | 9754.77(2754.59,24914.68) | 28.94(8.42,73.18) |  | 4101.95(1309.33,8838.82) | 24.03(7.18,52.52) |  | -0.55(-0.81,-0.28) |
| Democratic Republic of the Congo | 599176.18(203890.13,1193633.05) | 687.95(239.1,1358.61) |  | 476926.1(165998.23,1011893.31) | 345.01(122.86,726.97) |  | -3.22(-3.6,-2.85) |
| Denmark | 15.07(11.49,20.03) | 0.24(0.19,0.32) |  | 11.49(8.51,15.75) | 0.15(0.11,0.22) |  | -1.59(-2.06,-1.12) |
| Djibouti | 2077.01(715.04,4258.51) | 215.72(79.13,429.19) |  | 2870.53(998.31,6036.23) | 177.96(65,372.12) |  | -0.93(-1.24,-0.62) |
| Dominica | 118.92(39.69,257.93) | 145.78(49.47,314.58) |  | 61.02(19.08,137.1) | 147.97(43.4,337.37) |  | 0.29(-0.02,0.59) |
| Dominican Republic | 15661.53(5076.33,34134.67) | 142.45(48.99,303.58) |  | 16756.78(5744.55,35391.48) | 152.94(52.49,323.06) |  | -0.49(-0.9,-0.09) |
| Ecuador | 21740.83(6559.42,48768.52) | 152.1(46.2,340.52) |  | 14987.37(4288.68,34341.05) | 88.95(25.43,203.69) |  | -1.97(-2.09,-1.84) |
| Egypt | 16332.52(4842.24,37497.79) | 18.26(5.52,41.74) |  | 10832.52(2810.6,26634.64) | 10.61(2.75,26.09) |  | -2.22(-2.41,-2.03) |
| El Salvador | 2291.76(704.59,5262.29) | 29.78(9.52,67.66) |  | 788.83(254.24,1792.07) | 14.42(4.59,32.87) |  | -3.12(-3.44,-2.81) |
| Equatorial Guinea | 7892.55(2513.54,17137.76) | 747.57(246.71,1607.39) |  | 13425.65(4762.04,27258.2) | 727.02(258.1,1474.34) |  | 0(-0.13,0.12) |
| Eritrea | 11110.68(3739,23413.58) | 206.96(78.12,423.57) |  | 9355.87(3162.76,19837.56) | 102.61(36.5,213.17) |  | -2.08(-2.48,-1.68) |
| Estonia | 112.47(6.57,146.85) | 5.85(0.39,7.6) |  | 1.75(1.24,2.68) | 0.12(0.08,0.19) |  | -9.61(-12.59,-6.54) |
| Eswatini | 10675.32(3848.33,21793.35) | 714.94(259.7,1445.68) |  | 3370.05(1116.07,7170.42) | 241.02(81.04,510.6) |  | -3.3(-3.65,-2.95) |
| Ethiopia | 954725.33(310317.06,2050125.94) | 849.19(289.56,1781.9) |  | 380077.75(128307.01,825700.91) | 223.33(78.59,478.9) |  | -5(-5.28,-4.72) |
| Fiji | 1155.63(375.64,2424.21) | 127.02(41.99,265.51) |  | 1051.4(312.69,2328.28) | 124.18(36.86,275.23) |  | -0.13(-0.32,0.06) |
| Finland | 24.95(15.25,30.93) | 0.41(0.27,0.5) |  | 13.29(9.48,18.06) | 0.2(0.14,0.28) |  | -1.79(-2.16,-1.43) |
| France | 444.38(364.44,526.69) | 0.65(0.54,0.76) |  | 190.39(148.47,253.76) | 0.23(0.17,0.31) |  | -3.05(-3.49,-2.61) |
| Gabon | 6741.62(2282.95,15213.33) | 368.56(129.01,818.23) |  | 3716.86(1192.91,8155.53) | 183.07(59.94,401.05) |  | -3.74(-4.29,-3.19) |
| Gambia | 7600.46(2336.08,16930.08) | 336.86(105.63,746.64) |  | 7577.97(2461.28,16812.07) | 223.5(72.88,495.32) |  | -2.38(-2.78,-1.98) |
| Georgia | 66.69(28.13,83.76) | 1.11(0.48,1.38) |  | 11.42(8.49,15.33) | 0.27(0.21,0.37) |  | -1.06(-3.1,1.02) |
| Germany | 795.29(563.01,934.86) | 0.76(0.57,0.89) |  | 297.44(234.66,385.69) | 0.25(0.19,0.34) |  | -3.54(-4.07,-3.01) |
| Ghana | 48861.85(15137.11,114385.94) | 173.04(54.83,401.82) |  | 66040.73(21567.5,146223.43) | 162.81(53.61,359.64) |  | -0.73(-1.25,-0.2) |
| Greece | 39.77(29.9,47.88) | 0.33(0.26,0.4) |  | 22.56(16.23,31.13) | 0.18(0.13,0.26) |  | -1.43(-1.78,-1.07) |
| Greenland | 6.88(2.27,14.96) | 12.22(4.22,26.17) |  | 3.79(1.45,8.43) | 9.12(3.08,21.07) |  | -0.91(-1.04,-0.79) |
| Grenada | 6.22(4.9,7.82) | 7.58(6.03,9.37) |  | 1.9(1.53,2.4) | 1.83(1.46,2.28) |  | -3.45(-3.92,-2.97) |
| Guam | 398.23(132.48,798.03) | 221.38(73.78,443.58) |  | 329.69(104.86,680.43) | 209.87(66.65,433.07) |  | -0.18(-0.24,-0.13) |
| Guatemala | 546.12(453.96,648.61) | 5.87(4.85,6.83) |  | 207.5(162.97,260.35) | 1.32(1.05,1.65) |  | -4.66(-5.01,-4.31) |
| Guinea | 53558.77(17099.87,114236.58) | 367.49(119.43,781.48) |  | 48326.3(14692.86,103699.68) | 213.61(65.49,458.08) |  | -2.5(-3.06,-1.94) |
| Guinea-Bissau | 19861.31(6034.82,43026.36) | 913.75(281.91,1972.04) |  | 11099.2(3514.24,24052.61) | 374.78(119.14,812.07) |  | -3.8(-4.96,-2.63) |
| Guyana | 218.97(156.89,308.7) | 23.45(17.32,32.07) |  | 38.12(25.8,54.42) | 5.21(3.5,7.56) |  | -3.78(-4.33,-3.24) |
| Haiti | 52095.79(17591.95,113282.46) | 455.05(159.77,978.32) |  | 65319.24(23958.5,133113.46) | 416.52(154.29,846) |  | -0.53(-0.96,-0.09) |
| Honduras | 2998.64(921.05,6684.22) | 36.01(11.82,79.14) |  | 2058.82(656.67,4675.66) | 18.63(6.13,41.74) |  | -3.6(-4.08,-3.12) |
| Hungary | 55.54(26.54,66.87) | 0.46(0.25,0.55) |  | 12.2(8.92,17.09) | 0.12(0.09,0.17) |  | -3.1(-4.19,-2.01) |
| Iceland | 2.06(1.36,2.45) | 0.77(0.51,0.92) |  | 0.76(0.54,1.27) | 0.19(0.14,0.3) |  | -4.46(-4.94,-3.98) |
| India | 1405372.07(447614.61,3111833.86) | 113.51(36.7,250.16) |  | 621529.6(198919.02,1350394.75) | 54.82(17.38,119.46) |  | -3.51(-4.42,-2.59) |
| Indonesia | 322929.03(97560.24,737817.93) | 143.03(43.93,325.37) |  | 320591.03(98809.8,705929.01) | 172.26(52.91,380.11) |  | 1.02(0.66,1.37) |
| Iran (Islamic Republic of) | 10742.51(3261.68,25829.06) | 12.74(4.01,30.39) |  | 5794.76(1985.69,12332.27) | 8.72(2.9,18.71) |  | -1.36(-1.63,-1.09) |
| Iraq | 9069.13(2706.35,20510.53) | 27.48(8.44,61.43) |  | 9868(3022.91,21721.43) | 21.44(6.6,47.16) |  | -0.71(-0.88,-0.53) |
| Ireland | 16.24(11.45,20.01) | 0.43(0.32,0.53) |  | 9.08(6.46,12.81) | 0.17(0.12,0.25) |  | -2.64(-2.98,-2.3) |
| Israel | 22.66(17.2,27.21) | 0.47(0.35,0.57) |  | 17.83(13.15,24.71) | 0.19(0.14,0.27) |  | -3.71(-4.42,-3) |
| Italy | 942.58(826.49,1121.01) | 1.57(1.39,1.77) |  | 402.29(305.64,542.27) | 0.48(0.37,0.62) |  | -4.66(-5.12,-4.19) |
| Jamaica | 76.2(59.27,96.57) | 3.18(2.54,3.96) |  | 46.07(33.41,61.96) | 1.74(1.26,2.35) |  | -0.54(-1.6,0.53) |
| Japan | 1178.17(961.33,1404.56) | 0.76(0.63,0.91) |  | 774.25(590.33,1010.63) | 0.39(0.3,0.52) |  | -2.09(-2.37,-1.81) |
| Jordan | 2435.26(726.97,5703.18) | 37.45(11.3,87.54) |  | 3504.09(1051.23,8095.41) | 29.63(8.89,68.46) |  | -0.95(-1.16,-0.74) |
| Kazakhstan | 412.25(336.25,493.78) | 2.58(2.12,3.05) |  | 110.34(82.49,159.6) | 0.63(0.47,0.89) |  | -6.6(-8.43,-4.75) |
| Kenya | 138638.1(44577.7,299648.24) | 298.01(100.5,635.1) |  | 82122.65(27173.35,176409.23) | 129.81(44.3,277.06) |  | -2.9(-3.23,-2.57) |
| Kiribati | 372.4(118.8,791.05) | 305.33(98.22,647.13) |  | 443.31(138.72,928.78) | 306.09(96.07,640.74) |  | 0.32(0.16,0.48) |
| Kuwait | 3.95(2.75,6.43) | 0.24(0.18,0.35) |  | 7.01(3.93,14.11) | 0.14(0.09,0.26) |  | -2.36(-3.08,-1.63) |
| Kyrgyzstan | 810.83(555.06,1280.5) | 13.81(9.54,21.58) |  | 31.81(23.57,44.99) | 0.47(0.35,0.68) |  | -13.02(-15.26,-10.73) |
| Lao People's Democratic Republic | 3790.11(1133.55,8489.23) | 49.67(15.37,109.37) |  | 2913.74(848.68,6808.29) | 35.83(10.56,83.26) |  | -1.2(-1.41,-0.99) |
| Latvia | 106.83(35.59,130.53) | 3.48(1.45,4.21) |  | 3.62(2.52,7.41) | 0.17(0.12,0.31) |  | -14.82(-16.98,-12.61) |
| Lebanon | 741.21(220.92,1682.6) | 14.26(4.37,32.15) |  | 667.44(186,1560.02) | 13.11(3.63,30.66) |  | -1(-1.29,-0.71) |
| Lesotho | 13107.39(4435.66,26865.98) | 464.69(161.3,937.81) |  | 5893.63(1874.87,13252.13) | 268.55(87.06,600.38) |  | -1.31(-1.56,-1.05) |
| Liberia | 31049.33(9891.47,67184.93) | 820.19(263.53,1768.06) |  | 45985.79(16543.96,90743.13) | 717.24(258.12,1415.6) |  | 0.33(-0.25,0.91) |
| Libya | 1227.02(372.08,2785.7) | 17.26(5.36,38.93) |  | 646.71(213.34,1418.55) | 16.1(5.11,35.63) |  | 0.46(0.11,0.81) |
| Lithuania | 79.76(21.4,97.58) | 2.07(0.65,2.51) |  | 3.55(2.52,5.62) | 0.12(0.09,0.19) |  | -9.82(-11.61,-7.99) |
| Luxembourg | 12.07(9.3,14.49) | 2.66(2.03,3.14) |  | 4.17(3.25,6.07) | 0.51(0.4,0.73) |  | -5.33(-6.04,-4.6) |
| Madagascar | 170196.32(59670.9,344793.91) | 689.17(245.07,1388.82) |  | 226553.85(82907.61,440407.98) | 558.61(208.2,1082.55) |  | -1.14(-1.61,-0.67) |
| Malawi | 96944.38(34409.79,196297.11) | 453.73(167.82,911.32) |  | 117171.73(42386.62,239573.23) | 451.95(166.05,919.71) |  | -0.75(-1.59,0.09) |
| Malaysia | 2912.77(924.55,6412.92) | 12.46(4.14,27.14) |  | 1632.26(592.93,3591.06) | 6.13(2.17,13.59) |  | -2.94(-3.26,-2.63) |
| Maldives | 110.56(32.96,251.02) | 27.86(9.3,60.77) |  | 55.87(18.36,122.95) | 13.61(4.38,30.04) |  | -2.21(-2.51,-1.92) |
| Mali | 102495.4(30618.73,230734.15) | 499.75(152.73,1120.9) |  | 184685.26(63933.5,372244.85) | 408.51(141.99,822.61) |  | -0.03(-0.39,0.32) |
| Malta | 1.18(0.95,1.53) | 0.31(0.25,0.4) |  | 0.58(0.4,0.93) | 0.13(0.09,0.21) |  | -2.75(-3.16,-2.34) |
| Marshall Islands | 176.12(55.93,361.99) | 242.32(77.3,496.88) |  | 140(46.42,283.12) | 235.61(78.18,476.11) |  | 0.15(-0.12,0.42) |
| Mauritania | 19132.11(6248.81,40613.52) | 455.56(151.33,964.02) |  | 13601.41(4392.26,29198.58) | 260.01(84.32,557.79) |  | -1.55(-1.79,-1.31) |
| Mauritius | 6.93(5.01,8.7) | 0.7(0.49,0.85) |  | 3.63(2.53,5.76) | 0.27(0.19,0.43) |  | -1(-2.18,0.2) |
| Mexico | 5614.61(4912.54,6351.61) | 5.73(5.09,6.37) |  | 1598.98(1353.46,1872.16) | 1.31(1.12,1.53) |  | -4.24(-5.1,-3.38) |
| Micronesia (Federated States of) | 564.89(184.46,1222.67) | 339.62(112.04,732.92) |  | 231.44(84.34,447.91) | 246.24(89.71,476.63) |  | -0.89(-1.09,-0.7) |
| Monaco | 1.63(0.61,3.57) | 11.12(3.56,25.83) |  | 1.2(0.46,2.54) | 7.1(2.38,15.86) |  | -1.71(-1.85,-1.57) |
| Mongolia | 4241.39(1145.65,9484.12) | 121.73(33,272.18) |  | 7120.11(1874,15187.13) | 183.81(48.45,391.89) |  | 2.38(1.77,3) |
| Montenegro | 71.25(22.48,162.59) | 14.52(4.5,33.39) |  | 40.13(13.79,86.12) | 11.72(3.73,25.93) |  | -0.89(-1.02,-0.76) |
| Morocco | 38232.4(11784.93,86657.46) | 101.7(31.48,230.26) |  | 18855.77(5286.89,43755.37) | 64.84(18.16,150.6) |  | -3.93(-5.02,-2.83) |
| Mozambique | 381955.93(134882.67,780203.55) | 1408.78(500.27,2864.53) |  | 218974.75(76585.82,454446.97) | 418.46(150.26,864.12) |  | -4.01(-4.32,-3.7) |
| Myanmar | 45381.18(13218.19,106524.18) | 72.95(21.87,170.29) |  | 44628.27(14400.9,97944.16) | 89.1(28.72,195.65) |  | 0.7(0.48,0.93) |
| Namibia | 7367.04(2342.53,15659.59) | 325.17(106.75,681.78) |  | 6251.05(2073.48,13116.92) | 209.72(70.38,438.97) |  | -2.99(-3.9,-2.07) |
| Nauru | 35.96(11.13,81.88) | 181.01(56.51,411.33) |  | 32.36(9.82,72.08) | 226.96(69.25,505.27) |  | 0.98(0.69,1.26) |
| Nepal | 60724.74(18083.9,136843.79) | 160.05(49.2,358) |  | 36626.61(11089.19,78633.06) | 124.1(37.64,266.4) |  | -1.02(-1.1,-0.94) |
| Netherlands | 136.37(75.68,161.37) | 0.76(0.44,0.89) |  | 54.33(41.57,71.45) | 0.23(0.18,0.32) |  | -4.26(-4.92,-3.6) |
| New Zealand | 16.97(12.09,21.1) | 0.46(0.33,0.57) |  | 16.05(11.88,21.1) | 0.27(0.2,0.37) |  | -1.06(-1.62,-0.5) |
| Nicaragua | 2705.4(825.02,5880.82) | 40.1(12.77,86.36) |  | 867.15(292.38,1855.83) | 13.75(4.69,29.34) |  | -5.01(-6.08,-3.93) |
| Niger | 14664.26(4798.23,32413.93) | 74.26(26.73,161.12) |  | 16728.96(5583,35986.95) | 31.66(11.16,67.19) |  | -3.98(-4.52,-3.43) |
| Nigeria | 378746.05(115870.87,833153.4) | 196.6(61.18,430.33) |  | 779214.85(248649.87,1780486.93) | 218.12(70.01,497.51) |  | 0.95(0.6,1.3) |
| Niue | 5.3(1.66,11.58) | 202.13(63.29,441.2) |  | 2.79(0.91,6.12) | 226.93(73.47,498.64) |  | 0.58(0.48,0.68) |
| North Macedonia | 228.9(67.58,520.95) | 13.48(3.94,30.76) |  | 132.09(41.56,296.65) | 11.96(3.56,27.21) |  | -0.22(-0.43,-0.02) |
| Northern Mariana Islands | 108.05(35.76,227.51) | 179.61(59.64,377.61) |  | 42.47(14.54,83.97) | 178.88(60.5,354.81) |  | 0.45(0.21,0.7) |
| Norway | 42.5(31.13,67.6) | 0.81(0.6,1.2) |  | 97.82(58.37,120.45) | 1.2(0.79,1.48) |  | 1.81(1.57,2.05) |
| Oman | 277.14(85.19,644.97) | 8.9(2.81,20.44) |  | 184.43(65.59,404.59) | 4.77(1.61,10.61) |  | -3.22(-3.61,-2.82) |
| Pakistan | 422627.28(121430.38,962529.12) | 186.14(54.59,421.63) |  | 416529.24(130476.54,918749.87) | 131.88(41.73,290.19) |  | -0.28(-0.69,0.14) |
| Palau | 37.93(12.11,82.33) | 214.89(68.64,466.37) |  | 24.01(8.57,47.09) | 263.83(94.05,516.65) |  | 1.08(0.93,1.24) |
| Palestine | 849.84(260.07,1912.11) | 19.08(5.95,42.44) |  | 963.3(294.47,2076.36) | 16.09(4.97,34.54) |  | -0.4(-0.54,-0.26) |
| Panama | 34.6(27.9,42.24) | 1.53(1.23,1.87) |  | 90.76(67.8,119.99) | 2.31(1.71,3.06) |  | 3.57(2.53,4.61) |
| Papua New Guinea | 42028.58(14002.7,90060.22) | 595.93(200.64,1276.64) |  | 77691.62(24732.45,162318.8) | 499.49(159.63,1042.53) |  | -1.3(-1.58,-1.02) |
| Paraguay | 17740.26(5678.5,37157.74) | 289.74(93.59,606.1) |  | 19564.41(6944.2,39172.87) | 320.53(113.78,641.59) |  | 0.5(0.23,0.76) |
| Peru | 48263.29(14479.06,103807.57) | 153.37(46.57,328.76) |  | 16721.91(5122.22,38383.04) | 54.13(16.53,124.3) |  | -5.16(-5.59,-4.73) |
| Philippines | 69095.54(20958.12,151764.39) | 70.19(21.56,153.76) |  | 99215.63(29396.47,221511.22) | 78.14(23.29,174.32) |  | 0.28(0.07,0.48) |
| Poland | 463.48(302.37,542.41) | 1.15(0.77,1.33) |  | 202.08(150.37,261.55) | 0.39(0.29,0.5) |  | -3.45(-3.89,-3) |
| Portugal | 207.5(166.29,242.68) | 2.53(2.07,3) |  | 35.05(24.88,48.91) | 0.31(0.22,0.41) |  | -7.25(-9.16,-5.31) |
| Puerto Rico | 38.08(32.37,49.33) | 1.09(0.93,1.4) |  | 32.7(23.41,42.53) | 0.76(0.56,1) |  | -1.48(-2.27,-0.68) |
| Qatar | 117.68(33.82,268.17) | 23.31(6.74,53.04) |  | 229.69(71.88,500.51) | 16.78(4.99,37.18) |  | -1.55(-1.74,-1.36) |
| Republic of Korea | 5537.24(1795.24,12401.42) | 16.75(5.29,37.81) |  | 3994.05(1530.49,8238.01) | 18.61(6.15,40.05) |  | 1.1(0.84,1.37) |
| Republic of Moldova | 84.77(62.52,101.11) | 2.04(1.58,2.46) |  | 9.03(6.59,14.81) | 0.26(0.2,0.39) |  | -7.66(-8.56,-6.75) |
| Romania | 160.18(136.25,193.97) | 0.87(0.74,1.02) |  | 53.1(40.03,74) | 0.36(0.28,0.48) |  | -5.49(-7.81,-3.11) |
| Russian Federation | 3196.07(2870.17,3575.12) | 2.45(2.21,2.73) |  | 1241.98(1023.08,1506.2) | 1.05(0.88,1.24) |  | -5(-6.51,-3.47) |
| Rwanda | 34288.67(11438.88,74104.46) | 243.34(88.84,506.2) |  | 24730.39(8984.71,52694.61) | 149.52(55.72,317.01) |  | -1.55(-1.92,-1.18) |
| Saint Kitts and Nevis | 6.65(5.46,8.15) | 16.2(13.41,19.75) |  | 1.88(1.34,2.48) | 3.59(2.61,4.74) |  | -3.82(-4.64,-2.99) |
| Saint Lucia | 29.41(23.15,37.01) | 18.24(14.66,22.46) |  | 6.93(5.24,9.06) | 5.17(3.65,7.18) |  | -3.36(-3.93,-2.79) |
| Saint Vincent and the Grenadines | 53.1(38.47,71.62) | 47.12(33.49,62.16) |  | 7.09(5.44,9.28) | 6.96(5.2,9.26) |  | -4.1(-5.18,-3.01) |
| Samoa | 428.53(134.77,915.88) | 257.9(81.25,549.84) |  | 444.62(145.67,952.69) | 240.52(78.88,515.5) |  | -0.18(-0.52,0.17) |
| San Marino | 1.14(0.37,2.78) | 8.28(2.49,20.47) |  | 1.1(0.4,2.33) | 6.69(2.19,14.74) |  | -0.62(-0.86,-0.39) |
| Sao Tome and Principe | 423.93(131.42,927.85) | 193.12(61.08,421.4) |  | 278.34(86.61,616.28) | 123.63(38.52,273.48) |  | -1.98(-2.6,-1.34) |
| Saudi Arabia | 3201.71(976.24,7495.13) | 12.61(3.95,29.32) |  | 1278.46(446.82,2659.73) | 5.55(1.77,11.81) |  | -2.81(-3,-2.63) |
| Senegal | 20898.99(6926.39,44273.08) | 131.82(45.34,276.61) |  | 14974.02(4963.61,31752.08) | 68.19(22.91,143.86) |  | -3.81(-4.39,-3.23) |
| Serbia | 1046.06(308.49,2357.86) | 16.19(4.71,36.65) |  | 383.5(122.97,845.63) | 9.33(2.85,20.83) |  | -1.88(-2.08,-1.68) |
| Seychelles | 13.47(4.95,27.78) | 17.04(6.49,34.81) |  | 11(4.32,23.43) | 14.53(5.24,31.83) |  | -0.33(-0.45,-0.21) |
| Sierra Leone | 21303.55(6376.62,46442.27) | 291.11(88.21,631.98) |  | 22902.37(6865.93,51830.56) | 174.37(52.53,394.02) |  | -1.99(-2.24,-1.75) |
| Singapore | 80.57(30.93,97.26) | 3.41(1.14,4.15) |  | 28.96(20.27,39.21) | 0.39(0.28,0.52) |  | -6.26(-7.59,-4.9) |
| Slovakia | 437.54(138.63,971.14) | 11.31(3.47,25.3) |  | 259.01(85.7,569.53) | 9.1(2.68,20.48) |  | -0.52(-0.67,-0.37) |
| Slovenia | 3.53(2.37,4.76) | 0.18(0.13,0.23) |  | 1.56(1.04,2.62) | 0.08(0.06,0.13) |  | -2.38(-2.68,-2.07) |
| Solomon Islands | 5670.63(2001.47,11172.75) | 836.79(296.8,1645.2) |  | 9183.23(3831.34,15939.04) | 920.93(384.81,1597.21) |  | 0.97(0.63,1.32) |
| Somalia | 48307.06(16751.49,102349.56) | 354.63(132.05,724.3) |  | 110982.32(39234.47,229778.46) | 284.58(104.67,571.49) |  | 0.04(-0.41,0.5) |
| South Africa | 315299.83(111843.03,637171.18) | 683(244.34,1376.4) |  | 200630.48(67832.56,407956.58) | 404.41(136.24,822.68) |  | -1.77(-2.27,-1.27) |
| South Sudan | 75355.66(24967.3,164611.24) | 658.08(229.06,1416.7) |  | 95068.41(29103.03,211490.91) | 564.29(176.91,1247.54) |  | 0.69(0.37,1.02) |
| Spain | 1061.55(789.59,1239.06) | 2.54(1.86,2.94) |  | 187.71(147.65,252.75) | 0.32(0.25,0.42) |  | -6.84(-7.67,-6) |
| Sri Lanka | 1942.47(653.23,4178.11) | 11.36(3.87,24.29) |  | 928.83(329.14,2052.34) | 6.13(2.02,13.97) |  | -2.6(-3.04,-2.16) |
| Sudan | 72906.24(20028.42,175624) | 159.25(44.01,382.45) |  | 88640.56(24919.89,213394.41) | 157.9(44.49,379.81) |  | -1.03(-1.54,-0.52) |
| Suriname | 852.93(256.63,1895.6) | 196.29(59.52,435.51) |  | 549.12(159.77,1235.35) | 126.36(36.3,285.08) |  | -1.73(-1.87,-1.6) |
| Sweden | 36.77(17.72,46.15) | 0.34(0.19,0.44) |  | 17.61(12.2,26.49) | 0.16(0.11,0.24) |  | -2.08(-2.48,-1.67) |
| Switzerland | 42.84(31.58,55.47) | 0.49(0.37,0.64) |  | 43.4(32.19,55.88) | 0.35(0.27,0.45) |  | -1.18(-1.48,-0.89) |
| Syrian Arab Republic | 6535.04(1843.98,16487.91) | 27.52(8.08,68.7) |  | 1818.68(587.42,3916.72) | 16.06(5.12,34.76) |  | -1.01(-1.31,-0.7) |
| Taiwan (Province of China) | 208.77(170.44,256.49) | 1.14(0.94,1.39) |  | 317.89(238.81,411.53) | 0.98(0.75,1.26) |  | -0.5(-0.99,-0.01) |
| Tajikistan | 1878.17(713.71,3894.43) | 23.57(11.04,44.08) |  | 1546.48(666.83,3071.56) | 14.14(6.69,26.88) |  | -2.86(-3.37,-2.35) |
| Thailand | 13324.59(4357.35,29399.8) | 25.94(8.45,57.43) |  | 2239.57(942.1,4510.47) | 7.17(2.62,15.25) |  | -6.92(-8.21,-5.61) |
| Timor-Leste | 1332.75(402.7,3019.04) | 79.93(24.88,179.45) |  | 1075.48(347.99,2408.44) | 59.11(19.3,131.8) |  | -1.14(-1.28,-1) |
| Togo | 14554.22(4503.81,33096.08) | 190.89(61.26,430.54) |  | 9448.71(3010.84,20982.01) | 84.17(27.19,186.55) |  | -5.3(-6.16,-4.43) |
| Tokelau | 5.5(1.75,11.46) | 191.9(61.62,399.72) |  | 2.48(0.85,4.9) | 139.05(47.58,274.47) |  | -0.83(-1.04,-0.61) |
| Tonga | 249.68(78.98,547.2) | 199.44(63.27,436.64) |  | 180.11(56.85,378.3) | 159.65(50.53,335.12) |  | -0.89(-1.05,-0.73) |
| Trinidad and Tobago | 6.06(4.92,7.89) | 0.58(0.47,0.74) |  | 9.51(7.32,12.42) | 0.6(0.46,0.79) |  | 0.24(-0.63,1.13) |
| Tunisia | 3059.28(870.07,6954.42) | 27.02(7.76,61.29) |  | 1089.16(357.16,2372.94) | 13.3(4.27,29.17) |  | -2.27(-2.47,-2.08) |
| Turkey | 16580.85(4821.38,38155.46) | 21.19(6.23,48.63) |  | 5304.35(1693.47,12035.77) | 11(3.36,25.14) |  | -2.23(-2.34,-2.13) |
| Turkmenistan | 55.98(27.58,66.58) | 1.84(0.74,2.2) |  | 10.97(7.52,17.04) | 0.21(0.15,0.34) |  | -9.14(-10.77,-7.47) |
| Tuvalu | 43.84(13.03,103.6) | 319.05(95.21,752.51) |  | 22.76(8,45.12) | 217.66(76.44,431.45) |  | -1.35(-1.51,-1.2) |
| Uganda | 165020.02(53562.67,359877.8) | 388.7(129.35,840.76) |  | 531597.33(203520.98,1034508.34) | 712.32(273.83,1383.54) |  | 1.31(0.71,1.91) |
| Ukraine | 3173.13(950.63,3724.43) | 4.88(1.48,5.73) |  | 228.36(149.24,796.32) | 0.44(0.29,1.37) |  | -15.06(-17.89,-12.14) |
| United Arab Emirates | 239.71(75.15,556.8) | 10.81(3.44,25.04) |  | 174.72(90.66,325.3) | 4.72(1.78,9.93) |  | -4.09(-4.71,-3.48) |
| United Kingdom | 4723.58(1618.26,10428.74) | 11.76(3.65,26.59) |  | 4328.53(1416.58,9188) | 11.07(3.36,23.79) |  | 1.09(0.67,1.52) |
| United Republic of Tanzania | 465365.29(171414.09,921182.7) | 824.81(309.93,1622.92) |  | 338311.24(122185.92,680853.41) | 348.27(126.73,696.9) |  | -3.36(-3.75,-2.98) |
| United States of America | 5017.23(4109.32,6164.03) | 1.82(1.52,2.21) |  | 4527.24(3537.22,5721.52) | 1.03(0.82,1.28) |  | -2.26(-2.5,-2.01) |
| United States Virgin Islands | 118.08(39.16,252.02) | 109.01(36.29,232.22) |  | 47.62(17.27,102.97) | 73.79(24.19,164.38) |  | -1.36(-1.42,-1.3) |
| Uruguay | 744.76(548.76,969.28) | 26.3(19.52,34.75) |  | 29.84(23.7,38.6) | 0.97(0.75,1.29) |  | -11.7(-13.54,-9.82) |
| Uzbekistan | 560.56(186.73,664.28) | 3.45(1.04,4.12) |  | 60.22(39.26,149.8) | 0.18(0.11,0.48) |  | -13.9(-15.69,-12.06) |
| Vanuatu | 880.71(295.25,1821.9) | 323.96(108.88,668.7) |  | 1534.11(542.21,2964.32) | 414.9(147.3,801) |  | 0.41(0.29,0.54) |
| Venezuela (Bolivarian Republic of) | 1447.23(1166.18,1794.4) | 6.08(4.99,7.4) |  | 1218.45(843.24,1716.39) | 4.89(3.32,7) |  | -1.71(-2.46,-0.95) |
| Viet Nam | 39170.23(11326.1,90854.89) | 44.82(13.27,103.94) |  | 20034.02(6271.47,43214.66) | 30.82(9.52,66.76) |  | -1.87(-2.02,-1.71) |
| Yemen | 25875.93(7881.02,63488.91) | 81.47(25.11,199.59) |  | 35405.72(10867.35,80377.68) | 79.52(24.6,180.08) |  | 0.04(-0.13,0.22) |
| Zambia | 99263.09(35073.78,196864.84) | 559.97(202.15,1103.83) |  | 119224.83(43156.04,242727.87) | 410.5(149.61,832.84) |  | -1.52(-1.78,-1.26) |
| Zimbabwe | 35937.33(11363.5,76156.8) | 202.08(66.29,422.55) |  | 37376.25(12949.55,76939.5) | 178.65(63,367.56) |  | -0.25(-0.42,-0.09) |

**Note:** disability adjusted life-years (DALY), estimated annual percentage change (EAPC), 95% uncertainty interval (UI), 95% confidence interval (CI), social development index (SDI).

**Supplementary Figure 1** The change trends of death cases, ASDR and EAPC among different SDI quintiles


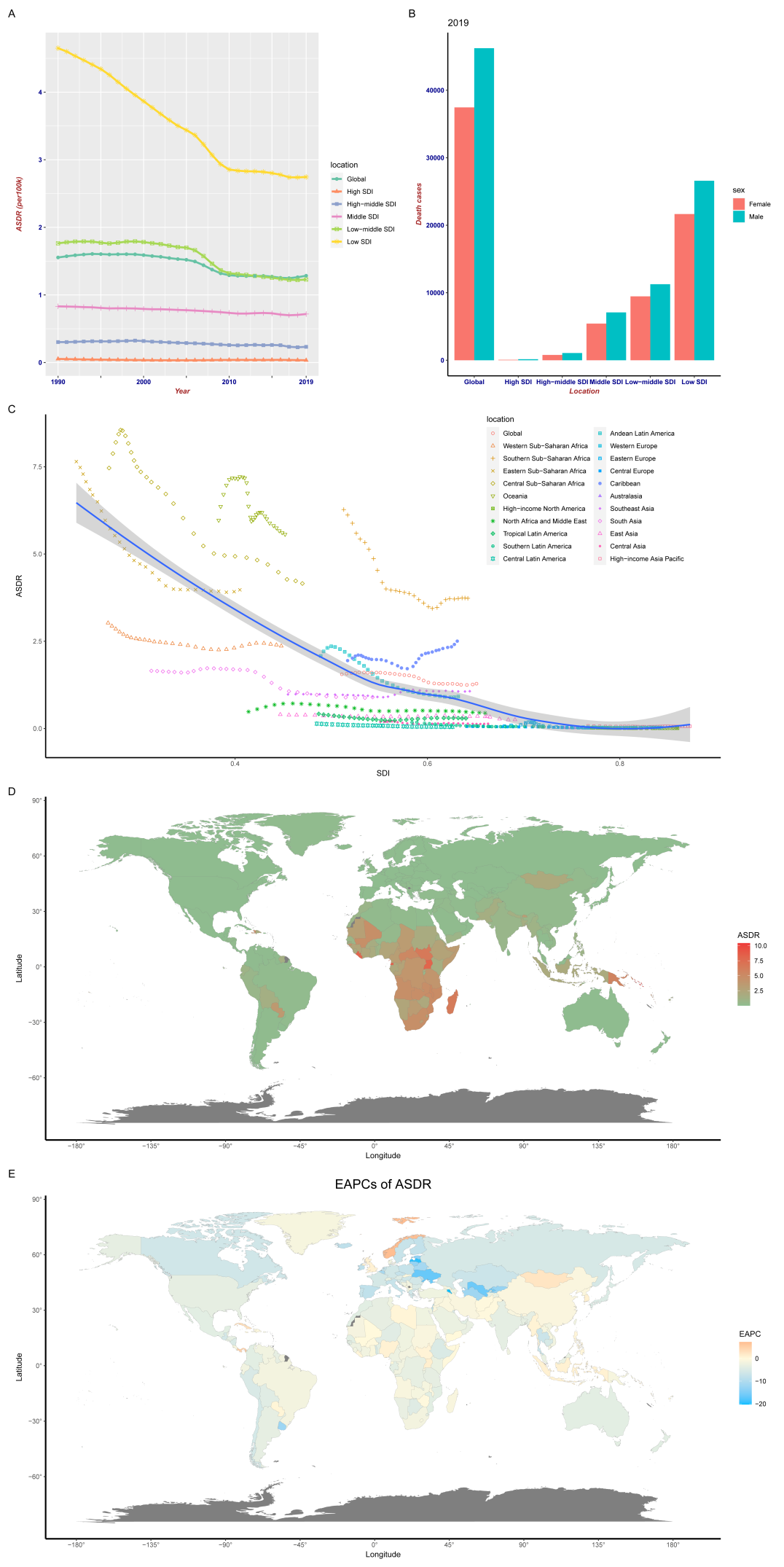


**Note:** A: Variation trend of ASDR in different SDI over time. B: Death cases of different SDI. C: The correlation between SDI and ASDR among 21 regions. D: ASDR in map. E: EAPC in map.

**Supplementary Figure 2** The change trends of DALYs, age−standardized DALY rate and EAPC among different SDI quintiles


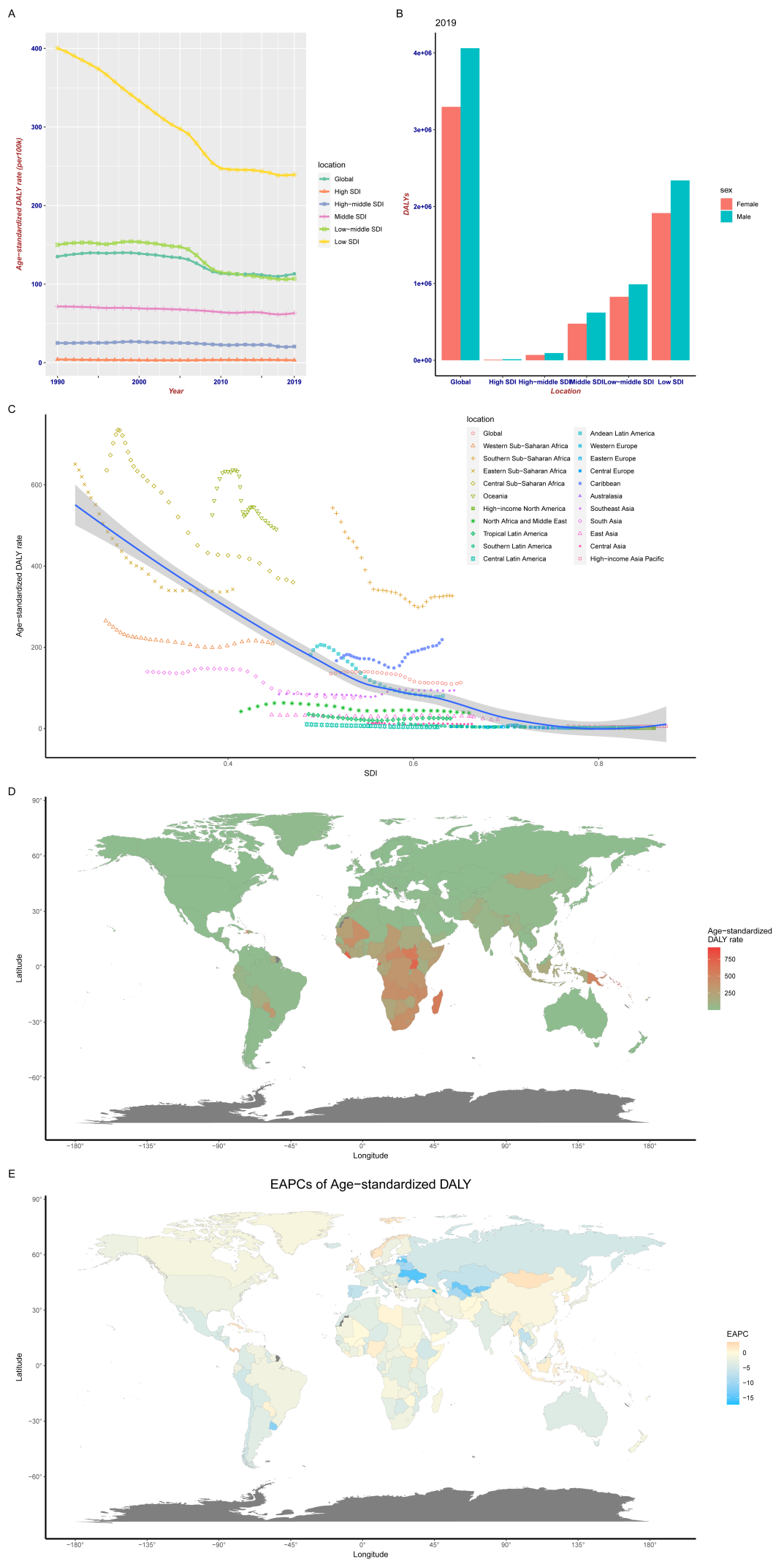


**Note:** A: Variation trend of age−standardized DALY rate in different SDI over time. B: DALYs of different SDI. C: The correlation between SDI and age−standardized DALY rate among 21 regions. D: Age−standardized DALY rate in map. E: EAPC in map.

**Supplementary Figure 3** The correlation between ASIR, ASDR, age−standardized DALY in 2019 and SDI, and the correlation between EAPCs and ASIR, ASDR, DALY in 1990.


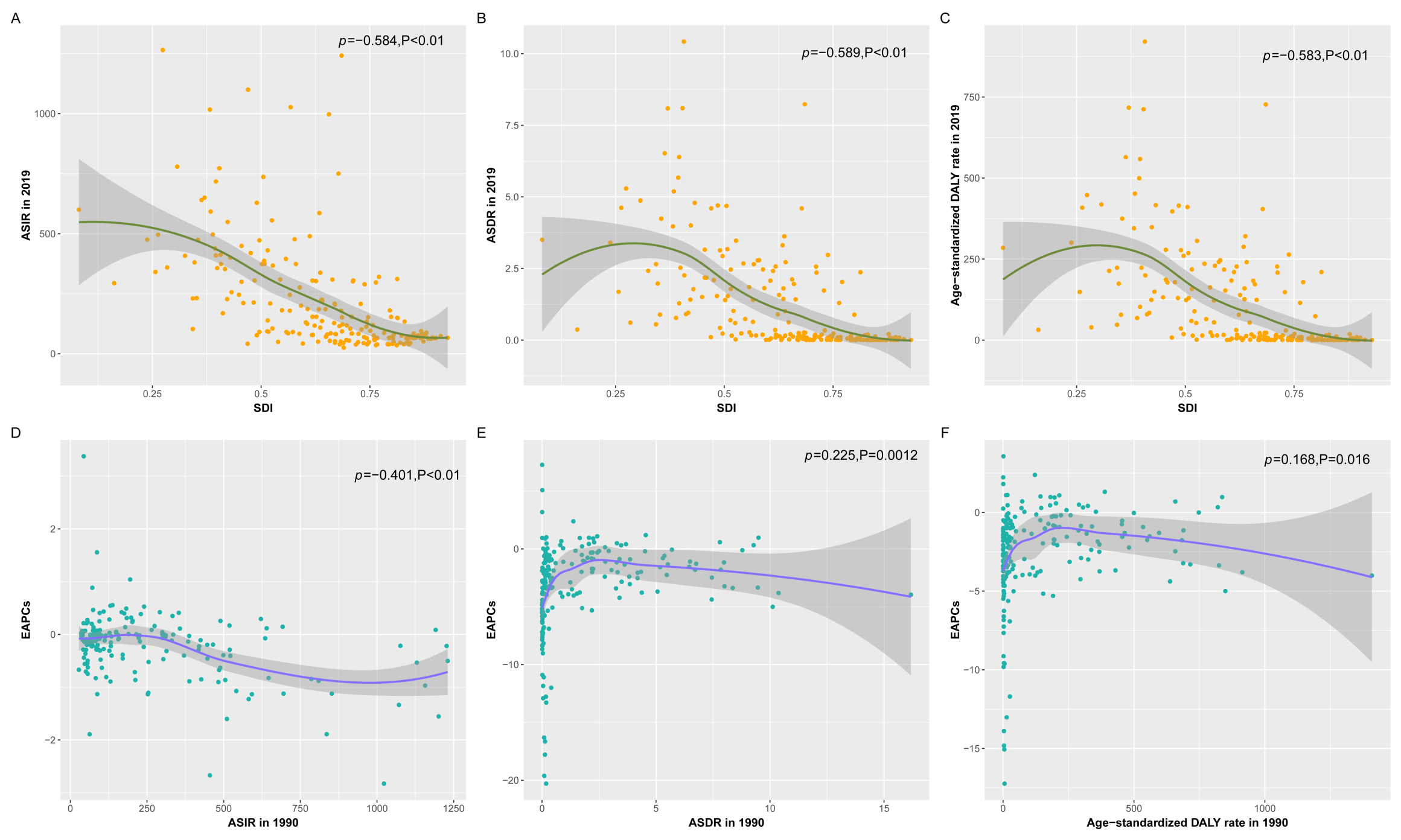


**Note:** A: The correlation between ASIR in 2019 and SDI. B: The correlation between ASDR in 2019 and SDI. C: The correlation between age−standardized DALY rate and SDI. D: The correlation between EAPCs and ASIR in 1990. E: The correlation between EAPCs and ASDR in 1990. F: The correlation between EAPCs and age−standardized DALY rate in 1990.
